# Emerging roles of extracellular vesicle-associated non-coding RNAs in hypoxia: Insights from cancer, myocardial infarction and ischemic stroke

**DOI:** 10.7150/thno.73931

**Published:** 2022-07-18

**Authors:** Dirk M. Hermann, Wenqiang Xin, Mathias Bähr, Bernd Giebel, Thorsten R. Doeppner

**Affiliations:** 1 Department of Neurology, University Hospital Essen, University of Duisburg-Essen, Essen, Germany; 2 Department of Neurology, University of Göttingen Medical School, Göttingen, Germany; 3 Institute of Transfusion Medicine, University Hospital Essen, University of Duisburg-Essen, Essen, Germany; 4 Research Institute for Health Sciences and Technologies (SABITA), Medipol University, Istanbul, Turkey; 5 Department of Anatomy and Cell Biology, Medical University of Varna, Varna, Bulgaria; 6 Department of Neurology, University of Giessen Medical School, Giessen, Germany

## Abstract

Hypoxia is a central pathophysiological component in cancer, myocardial infarction and ischemic stroke, which represent the most common medical conditions resulting in long-term disability and death. Recent evidence suggests common signaling pathways in these diverse settings mediated by non-coding RNAs (ncRNAs), which are packaged in extracellular vesicles (EVs) protecting ncRNAs from degradation. EVs are a heterogeneous group of lipid bilayer-covered vesicles released from virtually all cells, which have important roles in intercellular communication. Recent studies pointed out that ncRNAs including long non-coding RNAs (lncRNAs) and microRNAs (miRNAs) are selectively sorted into EVs, modulating specific aspects of cancer development, namely cell proliferation, migration, invasion, angiogenesis, immune tolerance or drug resistance, under conditions of hypoxia in recipient cells. In myocardial infarction and stroke, ncRNAs shuttled via EVs have been shown to control tissue survival and remodeling post-hypoxia by regulating cell injury, inflammatory responses, angiogenesis, neurogenesis or neuronal plasticity. This review discusses recent evidence on EV-associated ncRNAs in hypoxic cancer, myocardial infarction and stroke, discussing their cellular origin, biological function and disease significance. The emerging concept of lncRNA-circular RNA/ miRNA/ mRNA networks is outlined, upon which ncRNAs synergistically respond to hypoxia in order to modify disease responses. Particular notion is given to ncRNAs participating in at least two of the three conditions, which revealed a large degree of overlaps across pathophysiological conditions. Possible roles of EV-ncRNAs as therapeutic products or theranostic markers are defined.

## Introduction

Hypoxia, a pathophysiological condition characterized by reduced tissue oxygen content, is a hallmark of a variety of pathophysiological conditions 1, 2. Among these conditions, cancer, myocardial infarction and ischemic stroke play an eminent role, since they represent the most prevalent causes of long-term disability and death in medicine 3. Hypoxia profoundly influences transcriptional responses in the affected cells, e.g., by the transcription factor hypoxia-inducible factor-1α (HIF1α), which is degraded under normoxic conditions by the von Hippel Lindau protein-mediated ubiquitin protease pathway but which is stabilized upon hypoxia 4, regulating a large variety of genes controlling cell proliferation, metabolism, survival and differentiation 5, 6. In cancer, HIF1α-dependent gene expression promotes the development of aggressive tumor phenotypes 1, 7, 8. In myocardial infarction and stroke, persistent hypoxia and ischemia compromise tissue remodeling and recovery 2, 9. The pathophysiological mechanisms underlying hypoxia in cancer, myocardial infarction and stroke are very different. The main cause of hypoxia in cancer tissue are proliferating tumor cells growing out from blood vessels, which often have irregular network characteristics with compromised blood supply 1, 8. On the contrary, the primary cause of hypoxia in myocardial infarction and stroke is reduced blood flow associated with vascular occlusions and atherosclerosis 2, 10. Despite obvious pathophysiological differences, the gene responses to hypoxia in these three pathologies exhibit a high degree of similarities. Similarities relate to protein-encoding RNAs and non-encoding RNAs (ncRNAs).

Resulting from gene expression changes, cellular biology and communication are fundamentally altered under conditions of hypoxia. To coordinate tissue responses, extracellular vesicles (EVs) are released from hypoxic cells 11, 12. Based on their size and cellular origin, EVs are regularly classified as exosomes (60 to 150 nm), microvesicles (100 to 1000 nm) and apoptotic bodies (typically larger than 500 nm) 11, 13. Exosomes originate from the late endosomal compartment, whereas microvesicles and apoptotic bodies are derived from plasma membrane. EV secretion is considered as an evolutionarily conserved process, which plays important roles in intercellular communication 13. For this purpose, EVs transfer a large variety of cargos, including proteins, RNA, DNA, bioactive metabolites and lipids. It is broadly assumed that all these molecule species can be delivered to recipient cells. The heterogenous group of ncRNAs, which are widely found in EV preparations, has received great interest in EV-mediated cell signaling, since ncRNAs profoundly regulate gene responses at the transcriptional, post-transcriptional or epigenetic levels by interacting with DNAs, RNAs or proteins 14, 15. ncRNAs are categorized by the number of nucleotides constituting RNAs. ncRNAs with less than 200 nucleotides are defined as small ncRNAs, which include microRNAs (miRNAs), small interfering RNAs and piwi-interacting RNAs, whereas long ncRNAs (lncRNAs), which also comprise circular RNAs (circRNAs), are composed of more than 200 nucleotides 14, 16. Although the precise subcellular source of these EVs is still a matter of discussion - some EV-ncRNAs arise from nucleus - 17, shuttled ncRNA transport via EVs has been proposed to regulate tissue responses to hypoxia 18. In light of their roles in disease processes, EV-derived ncRNAs might be promising disease biomarkers and even be considered as therapeutic tools 19-21.

The present review summarizes the latest literature regarding the role of EV-ncRNA contents in the progression of hypoxic tumors, myocardial infarction and stroke. Aspects regarding the cellular and subcellular source of EV-associated ncRNAs, their cellular targets and biological functions are evaluated. The possible utility of EV-associated ncRNAs as therapeutic products and theranostic biomarkers is discussed.

## 1. EVs and ncRNAs

### 1.1 The characteristics and cellular origins of EVs

EVs are constantly released from eukaryotic cells, archaea and bacteria. They consist of a phospholipid bilayer and are abundantly present in various body fluids including blood, cerebrospinal fluid, urine, breast milk, and lacrima 22, 23. EVs can be classified into subcategories based on their biogenesis, size and physicochemical properties. Exosomes with a size of 60-150 nm and a density of about 1.10-1.21 g/ml 24, 25 are formed as intraluminal vesicles (ILVs) by membrane budding of late endosomes, which are released into the extracellular space as multivesicular bodies (MVBs) by MVB fusion with the plasma membrane 26, 27. Microvesicles, which are 100-1000 nm in diameter with a density of about 1.04-1.07 g/ml 25, are released from the plasma membrane by plasma membrane budding 26, 28. Apoptotic bodies, which are typically larger than 500 nm in diameter, are formed during programmed cell death by plasma membrane budding. In cancer, extremely large vesicles called oncosomes are produced, which are considered to transfer oncogenic messages. Under pathophysiological conditions, apoptotic bodies or oncosomes may be more abundant in certain fluids than exosomes or microvesicles and thus confound EV analyses 29, 30. Whereas exosomes and microvesicles are suggested as 'safe containers' for cargos mediating cell-to-cell communication, apoptotic bodies are released for degradation during the disassembly of dying cells.

Importantly, not all EVs released from live cells are involved in cell communication. Live cells may also release vesicles through a cellular excretion machinery that do not aim at transmitting biological signals but are meant for remote degradation especially in the liver 15, 17. Apparently, such garbage vesicles may contain ncRNA cargos that lack cell signaling roles. Besides, apoptotic cells not only fragment into the larger apoptotic bodies, but also generate many EVs in the size of exosomes. When preparing EVs in the size range of exosomes, combinations of exosomes, small microvesicles, small apoptotic vesicles and other small EVs, including nuclear EVs, are enriched 31. To harmonize the nomenclature, the *International Society of Extracellular Vesicles* recommended to define all prepared vesicles independent of their origin as EVs; if they are all in the size of exosomes, the EVs might be termed more specifically as small EVs 32. Unfortunately, not all scientists and especially researchers from the industry adopted the nomenclature and use the term exosomes as a synonym for small EVs. The issue is further complicated by the fact that hardly any method allows isolation of EVs; regularly concentrated EVs contain a number of non-EV associated byproducts, e.g. blood-derived EV samples typically contain a high load of lipoproteins and urine samples frequently aggregates of Tamm-Horsfall protein also named uromodulin 33, 34. Furthermore, in addition to potentially contributing to functional impacts, byproducts in EV preparations may significantly influence EV quantification 35, 36. The reader should be aware that at least in most of the studies EVs were not isolated but rather enriched likely to contain a panel of different non-EV-associated byproducts.

### 1.2 The characteristics and associated functions of ncRNAs

Among the different EV cargos, ncRNAs are most systematically studied 37, 38. Despite their heterogeneity of origin and diversity of biological function, there is meanwhile broad evidence supporting a role of ncRNAs in coordinating tissue responses to injuries. Although more than 20,000 proteins are encoded by the human genome, they only account for approximately 20% of the whole genome 39. Emerging evidence demonstrates that both short ncRNAs and lncRNAs play a crucial role in the regulation of gene expression in numerous pathophysiological states 40.

miRNAs are single-strand RNAs, which typically are 21-23 nucleotides in size and belong to the family of short ncRNAs. Released from the nucleus as single-strand pre-miRNA hairpins, pre-miRNAs are processed to mature miRNAs in the cytosol via cleavage by the endonuclease Dicer 41. Together with Dicer and associated proteins, miRNAs form the RNA-induced silencing complex (RISC) 42. As part of the RISC, miRNAs interact with complementary gene sequences in the 3' untranslated region of target mRNA sequences, repressing gene expression by argonaute (AGO)-mediated mRNA cleavage, by mRNA poly(A) tail shortening that destabilizes the mRNA, or by interference with mRNA-ribosome interactions 43-45. The human genome contains >600 genes with robust evidence of miRNA functions 46. These miRNAs target >60% of all human genes 47. Thus, single miRNAs can have hundreds, sometimes >1000 mRNA targets 47. In many cases, these miRNAs moderately influence mRNA expression levels. Due to their multiple mRNA targets, the biological consequences of this action are profound.

In contrast to miRNAs, siRNAs are formed as double-strand RNAs in the nucleus which are typically longer than pre-miRNAs 48, 49. Following cleavage by Dicer, 21-24 nucleotide siRNAs result, which dissociate to single-strand siRNAs upon interaction with the RISC. As part of the RISC, these siRNAs scan complementary mRNA sequences 50. Unlike miRNAs, siRNAs have tight target specificity 48, 50. siRNA binding induces cleavage of these target mRNAs.

Among small ncRNAs, piwi-interacting RNAs (piRNAs) are the largest in size. Their length varies between animal species, it typically ranges from 26 to 31 nucleotides 51. piRNAs form complexes with piwi-AGO proteins capable of binding mRNAs and cleaving them 51.

lncRNAs are transcripts with more than 200 nucleotides that are not translated into protein 39. lncRNAs include intergenic and intronic ncRNAs, and may involve sense and antisense RNA sequences. Although the biological role has so far been shown only for a small lncRNA proportion, they control transcription and translation in multiple ways, namely as transcription coregulators, ligands to nuclear transcription repressors, activators of transcription factors, regulators of epigenetic modifications, assistants in DNA double-strand break repair, as well as mRNA processing, splicing, transport, translation, and degradation 52, 53. The recently described circRNAs display a circular covalently bonded structure associated with a higher tolerance to exonucleases 54. They serve as scaffolds for chromatin-modifying complexes, regulate gene transcription and mRNA splicing, and act as miRNA sponges 55, 56.

### 1.3 ncRNA loading into EVs

Although ncRNAs inside EVs originate from the transcriptome of their source cells, the composition of these ncRNAs differs from their source cell ncRNAs 57. Among ncRNAs enriched in EVs, miRNAs are most abundant 58, 59. Several studies analyzed the loading and sorting processes of ncRNAs into EVs, for which numerous signaling pathways have been shown to be involved 60-62. Recent work demonstrated that miRNA sorting into EVs is not a random but a highly regulated process 63, 64. miRNAs are characterized by a uridine or adenine residue at their 3'-end, which is important for their recognition by AGO2. miRNAs with an adenylated 3'-end are predominantly found in cells, whereas miRNAs with a uridylated 3'-end are sorted in EVs, as shown in RNA sequencing studies on human B cells and their EVs 63. These results suggest that posttranscriptional miRNA modifications, notably, 3'-end adenylation and uridylation, might play a pivotal role in EV packaging.

There is increasing evidence that ncRNA sorting into EVs critically depends on membrane lipid and, more specifically, sphingolipid metabolism. The lipid composition of the EV membrane resembles that of membrane microdomains, which are characterized by a high content of cholesterol, phosphatidyl choline, sphingomyelin and ceramide 65. Ceramide formation is controlled by neutral sphingomyelinase-2 and acid sphingomyelinase, which are localized in the cytosolic and luminal membrane leaflets, respectively, and hydrolytically cleave sphingomyelin to ceramide 66-68. Ceramide has unique biophysical properties, as it can self-associate through hydrogen bonding, providing the driving force that results in the coalescence of microscopic microdomains to a large-scale macrodomains 67 and the budding of ILVs from MVBs 65. It has been proposed that membrane microdomains act as platforms for MVB sorting and that ncRNAs integrating into these platforms exhibit specific nucleotide motifs differentially predisposing these ncRNAs for microdomain membrane interaction 62. Indeed, RNAs binding to microdomains possess a specific secondary structure that differs from other RNAs 69. Randomly structured RNA sequences revealed 20-fold lower affinity to the microdomain domains. In addition, specific nucleotide sequences appear to be required for enhanced affinity to phospholipid bilayers, and domains with membrane affinity have not been observed in random RNA sequences 61, 70. Specific exosome-sorting RNA motifs have been shown for both miRNAs (called EXOmotifs) and mRNAs 62.

Intracellular transfer of ncRNAs from the nucleus to other subcellular compartments involves RNA-binding proteins, from which larger ribonucleoprotein particles are formed ensuring traveling along the cytoskeleton 71. To date, more than 500 RNA-binding proteins have been reported, which consist of approximately 25% of the protein content of EVs 57, 72. Emerging evidence demonstrates that ncRNAs can be transmitted into MVBs for exosome packaging or to the plasma membrane for extracellular secretion. This transmission takes place in association with RNA binding proteins like heterogeneous nuclear ribonucleoproteins A2/B1 (hnRNPA2B1), YBX1, SYNCRIP, AGO2 and others 57. Villarroya et al. revealed that in T cells, hnRNPA2B1 can target a specific motif of numerous miRNAs and transmit them into the ILVs 73. SYNCRIP selectively loads hepatocyte miRNAs with a 4-nucleotide motif near the 3'-end 74, whereas YBX1 selectively carries miR-223 into HEK-293T cell EVs 75. Deletion of AGO2 in HEK293T cells significantly decreased miR-142-3p and miR-451 miRNAs in EVs 76, 77. Although a first picture of the mechanisms underlying ncRNA sorting is currently evolving, the extent to which these pathways are specific to certain ncRNA species in defined EV subsets is elusive. Future studies will have to evaluate how hypoxia regulates these packaging mechanisms.

### 1.4 Uptake of ncRNA-loaded EVs by recipient cells

**Figure 1:**
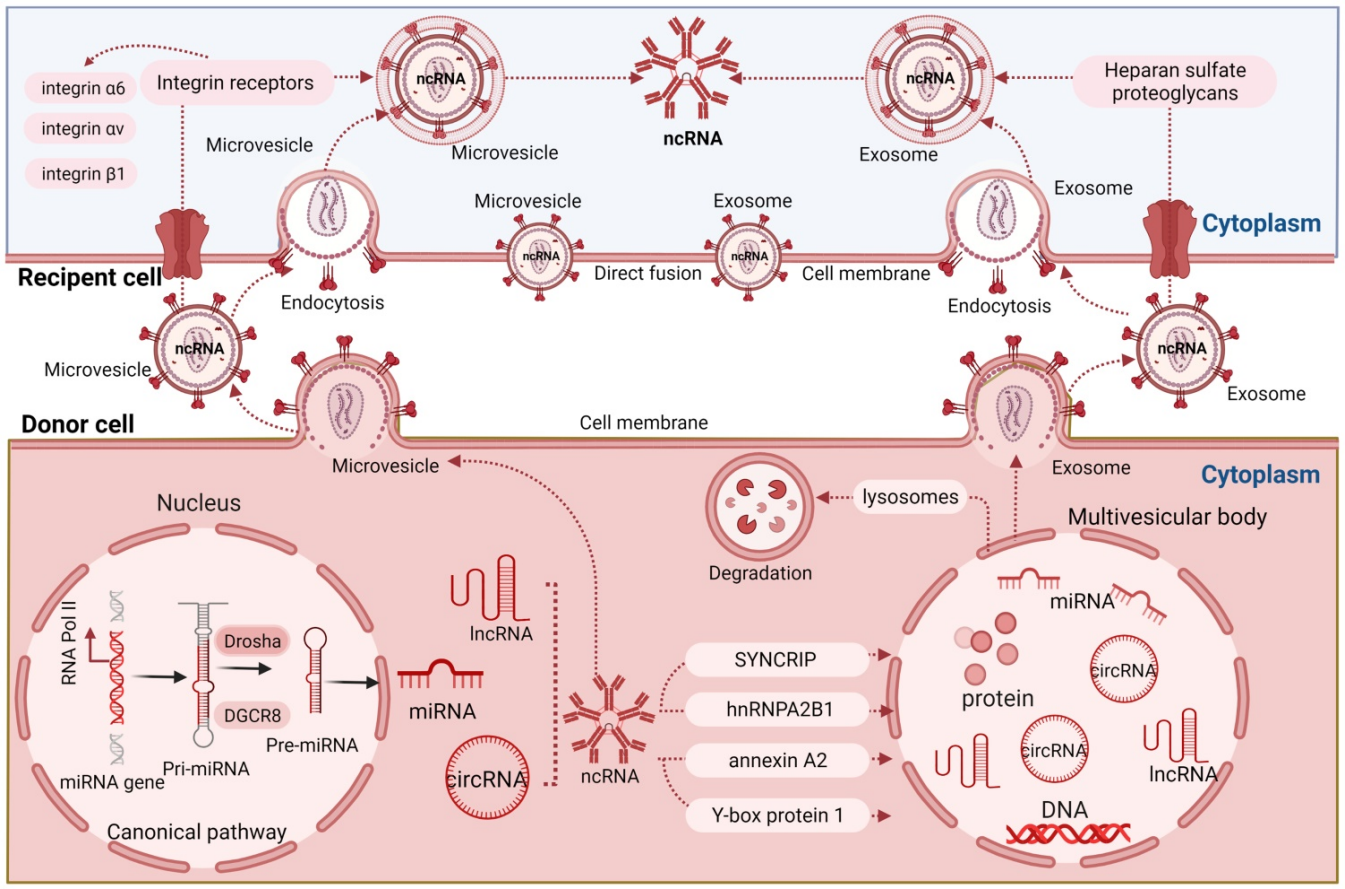
** Brief overview of mechanisms of cellular communication by EVs.** miRNAs are formed in the nucleus as pre-miRNAs that are processed to pre-miRNAs and mature miRNAs that are released into the cytosol. ncRNAs containing different RNA motifs are loaded into microvesicles and multivesicular bodies (MVBs) via different RNA-binding proteins. ILVs are formed within MVBs which are released into the extracellular space as exosomes. Recipient cells can take up EV-associated ncRNAs by direct fusion or endocytosis, both of which may be controlled by integrin receptor signaling.

To transmit ncRNAs into recipient cells, EVs may merge directly with the recipient cell by direct membrane fusion or be internalized through clathrin-mediated or caveolin-mediated endocytosis, phagocytosis or macropinocytosis 78. EV docking can be assimilated by recipient cells through directly targeting corresponding receptors on the plasma membrane, which in turn activates or inhibits associated signaling pathways 79. The receptors can be manipulated on the cell surface to increase EV uptake. For instance, enzymatic depletion or pharmacological inhibition of the extracellular matrix heparan sulfate proteoglycans on the plasma membrane has been found to promote the uptake of tumor-derived EVs by endocytosis 80. In the latter study, EV uptake was specifically inhibited by free heparan sulfate chains, whereas closely related chondroitin sulfate had no effect 80. Several integrin receptors have been shown to modulate EV uptake in a variety of cancers 57. As such, integrin receptors were shown to be enriched in cancer EVs compared to EVs obtained from benign epithelial cells 81. The total EV integrin levels, including the quantity of integrins α6, αv, and β1, correlated with tumor stage across a variety of epithelial cancers, while integrin α6 was prominently expressed on breast and ovarian progenitor cells 81, suggesting a role of these integrins in cellular EV uptake and the utility of EV integrins as potential theranostic markers. A crucial role in EV uptake might be related to phosphatidylserine, which is highly abundant on the surface of apoptotic cells but also present on a subpopulation of EVs 82, where phosphatidylserine considerably acts as 'eat me' signal by phagocytes 83. In mouse macrophages, the EV uptake is mediated via interaction of phosphatidylserine with T cell immunoglobulin and the mucin domain-containing protein 4 (Tim4) 83. Indeed, the delivery of anti-Tim4 antibody prevented EV uptake by thymic macrophages 83. Despite these emerging data, our current understanding of EV uptake mechanisms is still preliminary. Open questions remain about the endosomal escape of internalized EVs, which is required to deliver luminal EV cargo including ncRNAs into the cytosol of the EV recipient cells. In this context, it is interesting to note that even after successful EV uptake the generation of functional proteins from EV-derived mRNAs was negligible in recipient prostate cancer cells 57, 84. Presumably, these mRNAs could not be released from the endosomal system and thus were unable to reach the ribosomes. Hence, dissection of potential endosomal escape mechanisms is vital for understanding whether ncRNA mediate biological functions after EV uptake. It needs to be considered that EVs containing ncRNAs are taken up as cell nutrition. After EV internalization residual parts are degraded in the lysosome or excreted for digestion in remote cells or tissues, including the liver. Indeed, EV biodistribution studies detect the liver as very prominently labeled organ 85. A brief overview of mechanisms of communication between cells by EVs is shown in **Figure 1.**

### 1.5 Possible ncRNA artifacts associated with EV isolation and purification

In a variety of well-defined settings, authors could not confirm that ncRNAs and more specifically miRNAs are sorted into EVs 57. Certainly, results strongly depend on the EV preparation method. Originally and still often today, EVs are isolated by differential ultracentrifugation 86, which as stated before, also results in the preparation of many lipoprotein particles 87. Indeed, lipoproteins frequently contain miRNA-binding AGO proteins and thus can protect extracellular miRNAs 88. Bead capturing experiments using EVs obtained from mesenchymal stromal cells (MSCs) revealed that EVs recovered by cholera toxin b, a GM1 ganglioside ligand and membrane microdomain marker, contained many exosome markers but hardly any RNAs 17. In contrast, EVs captured by the globotriaosylceramide ligand shiga toxin b were abundant in nuclear markers and contained large amounts of RNAs 17. Interestingly, among the many studies using proteomic methods to analyse the composition of EVs, only one has reported the presence of AGO2 and none has detected Dicer 57. Apparently, the cellular source of EVs and physiological condition in which cells are raised decisively influence EV-ncRNA cargos. In the interpretation of ncRNA findings, possible artifacts related to EV isolation and purification carefully need to be considered.

## 2. Roles of EV-ncRNAs in the hypoxic tumor microenvironment

**Figure 2:**
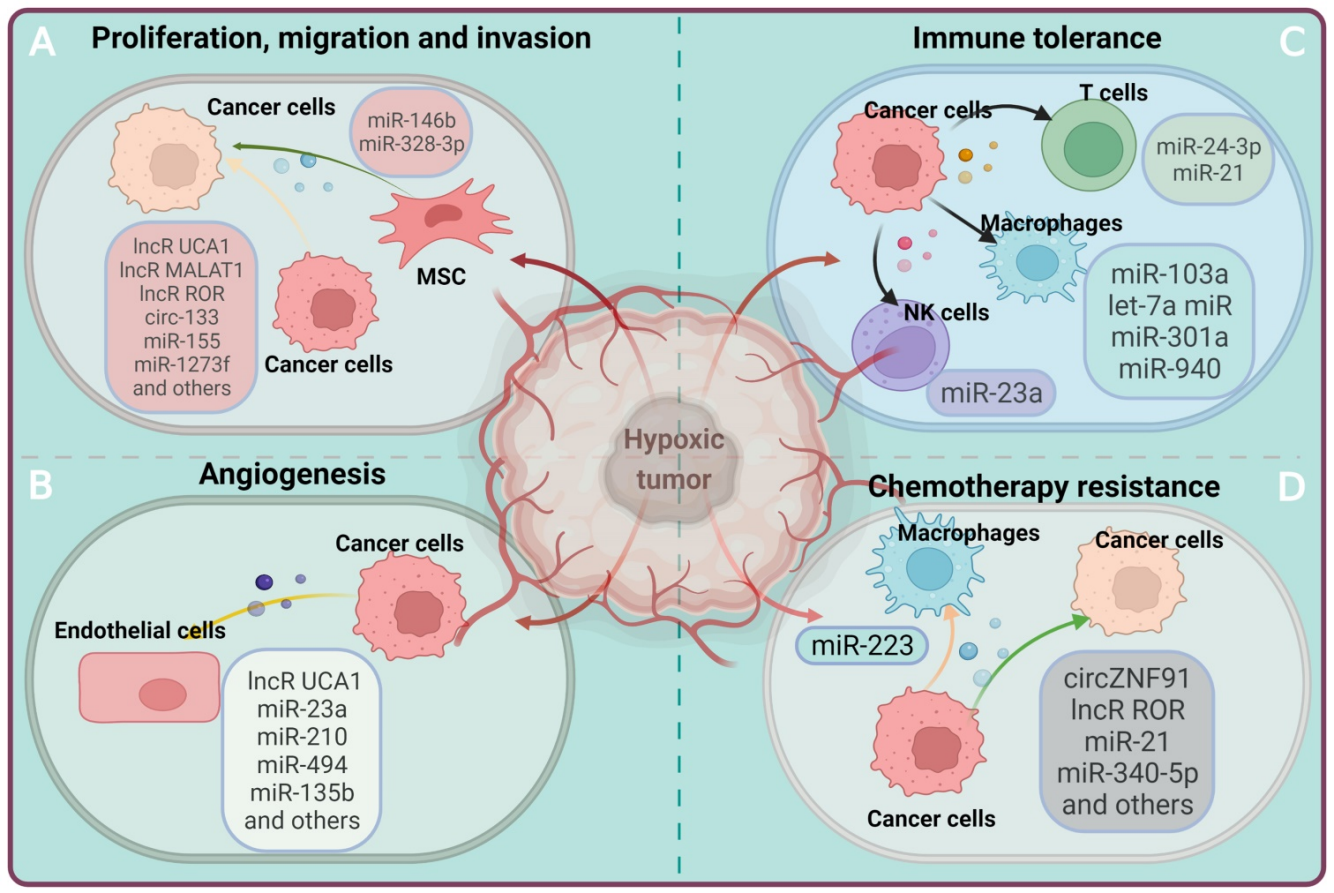
** The involvement of EV-ncRNAs derived from the hypoxic tumor in the regulation of the tumor microenvironment.** Hypoxic cancer cells can affect recipient cells by transferring ncRNAs via EVs, which, in turn, are taken up by recipient cells and modulate various biological processes including cell proliferation, immune tolerance, angiogenesis and drug resistance, thus facilitating tumor growth and progression.

Hypoxia is a key feature of solid tumors 89. Highly proliferating tumor cells outgrow the existing local blood supply, forming irregular vessel networks that cannot compensate for tissue oxygen needs 90, 91. Hence, tissue oxygen levels can drop below 2% in tumor masses, which has profound effects on the release and ncRNA contents of EVs 92. ncRNAs released from hypoxic tumor cells via EVs play an important role in creating a microenvironment that supports tumor growth 93-101. Notably, a large number of ncRNAs are increased in EV preparations obtained from hypoxic tumors. Several of these ncRNAs have been attributed to signal pathways associated with cell survival and proliferation, such as the FoxO pathway, the proteoglycans in cancer pathway, the HIF-1 signaling pathway or the mitogen-activated protein kinase (MAPK) pathway 11. The involvement of EV-derived ncRNAs in regulating tumor cell proliferation, angiogenesis, immunosuppression, drug resistance has been studied extensively in specific cancers under conditions of hypoxia 93-136, as outlined in **Table 1** and **Figure 2**. The main findings are summarized in the following.

### 2.1 Tumor cell proliferation, migration and invasion

Excessive cellular proliferation is a fundamental characteristic of cancer, which results from the activation of oncogenic signals that overrule the physiological inhibition of cell growth 137. EV-derived ncRNAs contribute to tumor cell proliferation, as has been brought into the spotlight recently. In EVs obtained from bladder carcinoma cells raised under hypoxic conditions, the content of urothelial carcinoma associated-1 (UCA1), a hypoxia-responsive lncRNA, was found to be enriched compared with EVs obtained from normoxic bladder carcinoma cells 102. UCA1 transfer via EVs obtained from hypoxic bladder carcinoma cells promoted tumor cell proliferation, migration, and invasion in recipient cells via mechanisms that involved epithelial-mesenchymal transition, a process relevant for cancer progression 102. The level of lncR-UCA1 in human serum-derived EVs of bladder carcinoma patients was higher than that in healthy control patients 102. Similarly, in hepatocellular carcinoma, lncRNA ROR accumulated in hypoxic tumor cell EVs was found to promote cancer growth by miR-145 downregulation and HIF1α stabilization 120. In non-small cell lung adenocarcinoma, lncRNA metastasis-associated lung adenocarcinoma transcript-1 (MALAT1) significantly increased tumor cell proliferation, migration, invasion, colony formation and glycolysis via mechanisms involving miR-515 and miR-613 sponging, followed by EEF2 and COMMD8 upregulation 123-125. In breast carcinoma, lncRNA nuclear enriched abundant transcript-1 (NEAT1) promoted tumor cell proliferation, migration, invasion and metastasis via miR-141-3p sponging and KLF12 upregulation 122. In colorectal carcinoma, hypoxic EV-associated circ-133 promoted tumor cell migration and metastasis via miR-133 sponging, GEF-H1 and RhoA elevation 136.

Besides lncRNAs, EV miRNAs have been involved in the regulation of tumor cell proliferation in hypoxia. In EVs derived from colorectal carcinoma cells, miR-361-3p was described to be enriched, when cells were cultivated under hypoxic compared with normoxic conditions 103. EV-mediated miR-361-3p delivery from hypoxic cells promoted tumor growth and suppressed tumor cell apoptosis in recipient cells by interaction with TRAF3 resulting in the activation of the NFκB pathway 103. Likewise, in EVs from hepatocellular carcinoma cells, miR-1273f was enriched when cells were raised under hypoxic compared with normoxic conditions 98. miR-1273f delivery via EVs obtained from hypoxic hepatocellular carcinoma cells was reported to increase miR-1273f levels in normoxic target cells, enhancing their proliferation by downregulating LHX6 expression 98. Studies in renal cell carcinoma and lung carcinoma noted roles of miR-155 and miR328-3p in hypoxic cancer proliferation via mechanisms involving inhibition of FOXO3 expression and activation 104 or inhibition of tumor suppressor NF2 targeted Hippo pathway activation 105, respectively. In oral squamous cell carcinoma and ovarian carcinoma, a role of EV miR-21 has been shown in hypoxic cancer cell proliferation, migration and invasion via HIF1α and HIF2α stabilization 95, 114, and in lung carcinoma, roles of miR-31-5p, miR-193-3p, miR-201-3p and miR-5100 were found via SATB2-revered epithelial-mesenchymal transition, MAPK/ERK1/2 activation and STAT3 activation 97, 121. In the latter studies, roles in the regulation of tumor cell proliferation, migration and invasion were shown for miRNAs associated with cancer cell EVs and MSC EVs.

### 2.2 Tumor-associated angiogenesis

Tumor growth vitally depends on tumor-associated angiogenesis, which involves a plethora of events such as the basal membrane degradation, endothelial proliferation, migration, tube formation and branching 138. EV-derived ncRNAs are claimed to facilitate tumor-associated angiogenesis under conditions of hypoxia. In this process, the lncRNA UCA1 again seems to play a prominent role. UCA1 was elevated in EVs obtained from hypoxic compared with normoxic pancreatic carcinoma cells and in serum-derived EVs from pancreatic carcinoma patients 106. In serum EVs of pancreatic carcinoma patients, UCA1 levels were associated with poor patient survival 106. Exposure of HUVECs to UCA1 enriched EVs promoted angiogenesis *in vitro* and *in vivo* by acting as a sponge for miR-96-5p that relieved the repressive effects of miR-96-5p on its target gene AMOTL2 106. In leukemia, miR-210 was upregulated in hypoxic compared with normoxic tumor cells and their EVs, inducing angiogenesis by downregulating EFNA3 expression 107. Angiogenesis was also facilitated by miR-23a-enriched EVs from hypoxic lung carcinoma cells, which induced tight junction breakdown, vascular permeability, transendothelial tumor cell migration and tumor growth 108. As underlying mechanism, the suppression of the miR-23a targets prolyl hydroxylase 1 and 2 (PHD1 and 2) that promoted HIF1α accumulation was identified 108. In lung carcinoma, the delivery of miR-494 enriched EVs derived from hypoxic tumor cells enhanced angiogenesis via PTEN, Akt and eNOS signaling 109. Studies in hypoxia-resistant multiple myeloma revealed that miR-135b enrichment in EVs enhanced angiogenesis under conditions of hypoxia via suppression of its target factor inhibiting HIF1 (FIH) 101. In colorectal carcinoma, tumor EV miR-25-3p promoted angiogenesis, vascular permeability and metastasis by downregulating KLF2 and KLF4 134.

### 2.3 Immune tolerance

Immune surveillance plays a central role in controlling tumor growth. To support its own growth, tumors can induce immune tolerance. In this process, ncRNAs transmitted via EVs are thought to be involved 18, 139. In nasopharyngeal carcinoma, tumor cells found to be enriched in miR-24-3p were shown to inhibit T cell proliferation and Th1 and Th17 differentiation by downregulating its target FGF11, thus increasing MAPK extracellular kinase (ERK)-1/2 activity, increasing signal transducer and activator of transcription (STAT)-1 and STAT3 activity and reducing STAT5 activity 110. In a study comparing a variety of tumor cells models including lung carcinoma cells, EVs from hypoxic tumor cells displayed elevated miR-210 and miR-23a abundance 111. The authors showed that miR-23a in hypoxic EVs downregulated CD107a expression in NK cells and thus lowered their antitumor response 111. In oral squamous cell carcinoma, elevated miR-21 in EVs of hypoxic tumor cells inhibited γδ T cell activation by regulating PTEN and PD-L1 119. Myeloid-derived suppressor cells (MDSCs) modulate the immunosuppressive tumor microenvironment by inhibiting T cell activation. A sequencing analysis of miRNAs from both hypoxic and normoxic glioma-derived EVs found that miR-10a and miR-21 induced upon hypoxia promoted MDSC expansion and activation by targeting RAR-related orphan receptor alpha (RORA) and phosphatase and tensin homolog (PTEN) 93. In lung carcinoma, melanoma, pancreatic carcinoma and ovarian carcinoma, NEAT1, miR-103a, miR-301a, miR-940 and let-7a were shown to regulate macrophage polarization towards an immunotolerant M2 phenotype via mechanisms involving the PTEN, PI3K/ Akt, STAT3 or IQGAP1 pathways, besides others 100, 116-118, 126. The induction of immune tolerance facilitates tumor growth.

### 2.4 Chemotherapy resistance

Acquired chemotherapy resistance in response repeated drug exposure is an essential factor that contributes to poor prognosis in cancer. Unraveling the underlying mechanisms is a prerequisite for developing novel cancer treatments. ncRNAs shuttled via EVs have recently been shown to contribute to chemotherapy resistance development. Microarray analysis of EV-derived circRNAs from hypoxic and normoxic pancreatic carcinoma cells displayed that circZNF91 was increased in EVs obtained from hypoxic pancreatic carcinoma cells 112. Overexpression of circZNF91 induced chemotherapy resistance in normoxic PC cells, while circZNF91 knockdown attenuated chemotherapy resistance by competitively targeting miR-23b-3p 112. In colorectal carcinoma, EV-associated circRNA ciRS-122 promoted glycolysis and induced oxaliplatin chemotherapy resistance through miR-122 sponging and PKM2 upregulation 135. In hepatocellular carcinoma, the hypoxia-responsive lncRNA ROR was highly abundant in tumor cells and their EVs 99. Incubation of tumor cells with lncRNA ROR-rich EVs induced chemotherapy-resistance to sorafenib 99. Interestingly, sorafenib similarly increased lncRNA ROR levels in tumor cells and their EVs, whereas siRNA-mediated lncRNA ROR knockdown restored chemotherapy responsiveness via mechanisms involving diminished CD133^+^ cells in response to transforming growth factor-β (TGFβ), a known stimulant inducing chemotherapy resistance 99. In non-small cell lung carcinoma cells, hypoxia increased miR-21 levels in tumor cells and their EVs, promoting cisplatin chemotherapy resistance of recipient cells by downregulating PTEN 94. Notably, high miR-21 levels in non-small cell lung carcinoma samples was associated with short survival in patients receiving chemotherapy, but not in patients not receiving chemotherapy 94. In hypoxic macrophages, elevated miR-223 levels were noted in EVs conferring chemoresistance in ovarian carcinoma cells; and miR-223 was shown to mediate this action via mechanisms involving PTEN downregulation and phosphatidylinositol-3 kinase (PI3K)/ Akt overactivation 113. The role of EV-shuttled ncRNAs supports a role as markers in diagnostics or theranostics in cancer.

## 3. Roles of EV-ncRNAs in myocardial infarction

**Figure 3:**
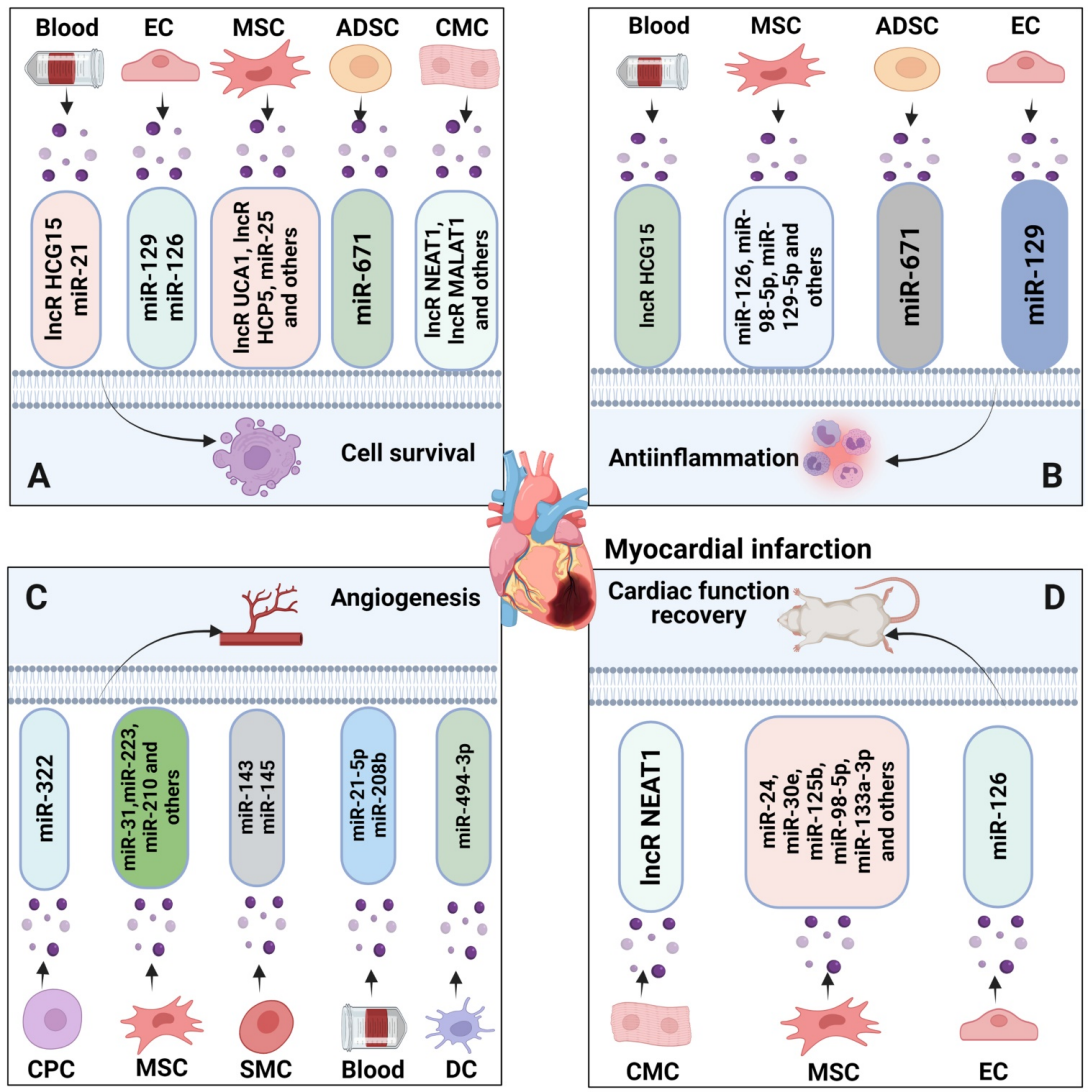
** The involvement of ncRNAs shuttled via EVs in myocardial infarction.** Different donor cells can impact recipient cells by transmitting ncRNAs via EVs, which can be taken up by recipient cells and alter various biological responses including cell survival, autophagy, inflammation and angiogenesis, thus regulating myocardial infarction progression and recovery. ADSC, adipose tissue-derived mesenchymal stromal cell; CMC, cardiomyocyte; CPC, cardiomyocyte precursor cell; DC, dendritic cell; EC, endothelial cell; SMC, smooth muscle cell.

The occlusion of a coronary artery in myocardial infarction results in a series of pathological events, among which necrosis, apoptosis, autophagy and inflammatory damage may ultimately lead to heart failure and death 2. Unlike in cancer, in which tumor development occurs progressively, myocardial infarction is an acute disorder characterized by an abrupt interruption of blood flow. Owing perhaps to the acute nature and severity of injury associated with myocardial infarction, several ncRNAs are downregulated in hypoxic-ischemic heart tissue in response to myocardial infarction, as previously shown for lncRNA HCP5, miR-21, miR-24, miR-30e, miR-98-5p, miR-150p, miR-185 and miR-212-5p 140-147, whereas other ncRNAs, such as lncRNA NEAT1 and miR-328-3p, are increased 148, 149. Delivery of EVs derived from bone marrow-derived mesenchymal stromal cells (MSCs) or patient blood seem to restore reduced ncRNA levels in ischemic heart tissue 140, 141, 143, 144, 147, 150. Suggestively, EV-ncRNAs play important roles in coordinating responses to myocardial injury, i.e., by modulating cardiomyocyte survival, inflammatory responses, angiogenesis and cardiac functional recovery 59, 151, 152. While EV-ncRNAs in hypoxic tumors have been widely studied in patient-derived cancer tissue, similar information from tissue obtained from patients with myocardial infarction is scarce due to the lack of access to histological tissue samples. Unlike in cancer, previous studies on myocardial infarction have mainly been conducted using tissues or cells without preexisting injury, which were experimentally exposed to hypoxia or ischemia 140-150, 153-180, as summarized in **Table 2** and **Figure 3** and further specified in the following.

### 3.1 Cell survival and injury

Studies in experimental models mimicking myocardial infarction imply that EV-associated lncRNAs and miRNAs can promote ischemic cardiomyocyte survival and cardiac function recovery. As such, UCA1, MALAT1, NEAT1, KLF3-AS1 and HCP5 lncRNAs shuttled via EVs from cardiomyocytes or MSCs were found to promote cardiomyocyte survival, inhibit cardiomyocyte autophagy and promote cardiac function recovery by mechanisms including miR-143, miR-92a, miR-23c, miR138-5p and miR-497 sponging 140, 149, 153-156. miRNA sponging potently increased the miRNA targets KLF2, STAT5B, SIRT1 and IGF1. Cardioprotective effects were demonstrated for several EV-miRNAs, namely miR-21, miR-25, miR-30e, miR-125b, miR-126, miR-146a, miR-185, miR-210, miR-212-5p, miR-338 and miR-671, which, collected from MSCs, cardiac progenitor cells, endothelial cells, endothelial progenitor cells or patient serum, promoted cardiomyocyte survival by downregulating miRNA targets including PDCD4, FASL, PTEN, LOX1, p53, BAK or SOCS2 141, 145-147, 150, 159, 162, 164, 165, 176-178, 181. As a consequence of EV administration, intrinsic and extrinsic cell death pathways were inhibited. Importantly, not all ncRNAs contained in EV samples protect against ischemic damage. Hence, EV-associated HCG15 lncRNA, miR-153-3p and miR-328-3p were found to exacerbate ischemic injury in myocardial infarction models via mechanisms involving NFκB/ p65 and p38 activation, PI3K/ Akt deactivation and caspase-3 activation, when obtained from ischemic cardiomyocytes, patient serum or MSCs 148, 167, 168.

### 3.2 Inflammation

The activation of pattern recognition receptors (PRRs) via damage-associated molecular patterns (DAMPs) may exacerbate ischemic cardiomyocyte injury via activation of the inflammasome, a multiprotein complex capable of cleaving and releasing proinflammatory IL1β levels, resulting in the activation of a proinflammatory type of programmed cell death called pyroptosis. EV-ncRNAs modulate inflammatory responses via a variety of miRNA targets. Hence, miR-98-5p and miR-129 transferred via endothelial cell EVs or MSC EVs reduced inflammasome activation in the ischemic myocardium by downregulating their miRNA target, the PRR TLR4, which in turn inhibited NFκB and the inflammasome component NLRP3 143, 158. Likewise, miR-146a and miR-671 shuttled via MSC EVs reduced TLR4, NFκB and SMAD2 signaling responses by downregulating their targets EGR1 and TGFBR2 150, 163. Patient serum-derived EV lncRNA HCG15, conversely, promoted IL1, IL6 and TNFα formation in ischemic myocardium via NFκB activation 167.

### 3.3 Angiogenesis

Vascular protective and angiogenic effects were shown for a variety of EV associated ncRNAs derived from cardiomyocytes, MSCs, cardiac progenitor cells, dendritic cells or patient serum, namely MALAT1 lncRNA, miR-21, miR-31, miR-126, miR-210, miR-223, miR-322, miR486-5p, miR-494-3p and miR-4732-3p in models of myocardial infarction 154, 157, 166, 168, 169, 171-173, 179, 180, 182. These ncRNAs were found to promote endothelial survival, proliferation, migration and tube formation by downregulating the miRNA targets including Krev interaction trapped protein-1 (KRIT1), matrix metalloproteinase-19 (MMP19), and FIH, thus inducing β-catenin activation, VEGF, CCND1, NOX2 or HIF1α elevation, respectively 154, 157, 166, 168, 169, 171-173, 180, 182. As a consequence of the enhanced tissue vascularization, fibrotic scar formation was reduced and cardiac function was enhanced 166, 170, 172, 180. In contrast, miR-153-3p delivered by MSC EVs compromised endothelial survival by downregulating its target angiopoietin-1 (ANGPT1) resulting in β-catenin activation, VEGFR2, PI3K/ Akt and eNOS deactivation 168, whereas miR-143 and miR-145 delivered by smooth muscle cell EVs reduced angiogenesis by downregulating their targets hexokinase-II (HKII) and integrin-β8 174. The cardioprotective, anti-inflammatory and angiogenic roles of EV-ncRNAs support a possible role of ncRNAs as diagnostic/theranostic markers and therapeutic targets in myocardial infarction.

## 4. Roles of EV-ncRNAs in ischemic stroke

**Figure 4:**
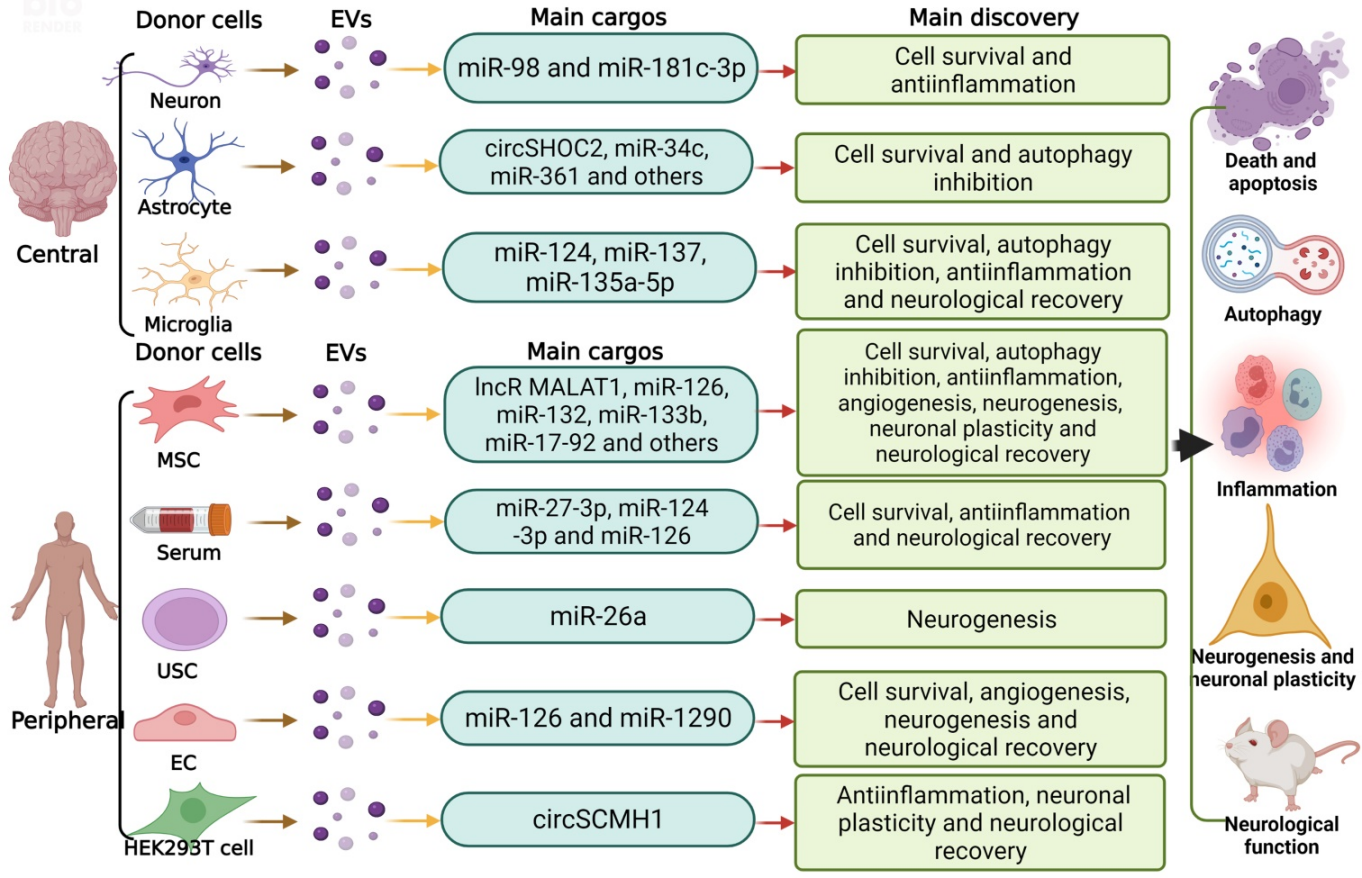
** The involvement of ncRNAs shuttled via EVs in ischemic stroke.** In the central and peripheral nervous system, different donor cells including neurons, microglia, astrocytes and MSCs can regulate recipient cells by transferring various EV-ncRNAs, modulating biological behaviors including neuronal survival, autophagy, inflammation, angiogenesis, neurogenesis and neuronal plasticity, thus modifying ischemic stroke progression and recovery. USC, umbilical cord-derived mesenchymal stromal cell; EC, endothelial cell

The occlusion of a cerebral artery affects the survival of brain neurons, glial cells and vascular cells. Among these different cells, the vulnerability of brain neurons is highest. Neuronal viability, structural connectivity and functional responses are vital for the recovery of lost neurological functions 183, 184. Yet, neurological recovery post-stroke critically depends on the successful restitution of vascular and glial functions. In the process of brain tissue remodeling, neurons, glial cells and vascular cells tightly interact with each other, preparing the stage so that successful functional recovery can occur 183. Similar to myocardial infarction, a variety of ncRNAs, namely circSCMH1, miR-124-3p, miR-126, miR-132, miR-221-3p and miR542-3p, are reduced in the ischemic brain and blood 185-191, whereas others, namely miR-98 and miR-494, are increased at defined time-points 192-194. Delivery of MSC-derived EVs can boost ncRNA levels in ischemic brain tissue 195-198. Although ischemic stroke and myocardial infarction have distinct pathophysiological features, they thus share common signaling pathways. Hence, EV-ncRNAs have vital roles in coordinating tissue responses to ischemic stroke in the acute and post-acute stroke setting, in which ncRNAs modulate neuronal survival, inflammatory responses, angiogenesis, neurogenesis and neuronal plasticity 151, 199. Similar to myocardial infarction, most studies on ischemic stroke have previously been performed using tissues or cells, which were experimentally exposed to hypoxia or ischemia 185-192, 195-198, 200-224, as summarized in **Table 3** and **Figure 4** and outlined in the following.

### 4.1 Cell survival and injury

Experimental stroke studies revealed that EV-ncRNAs can promote ischemic brain tissue survival in the acute stroke phase, reduce the development of brain atrophy in the chronic phase and enhance neurological recovery. Thus, EV samples obtained from hypoxic astrocytes that contained circSHOC2 lncRNA promoted neuronal survival via mechanisms involving miR-7670-3p sponging, resulting in SIRT1 overexpression 200. In HT22 neuronal cells, lncRNA MALAT1 shuttled via MSC EVs promoted neuronal survival via mechanisms including the recruitment of the serine-arginine-rich splice factor-2 (SRSF2), resulting in alternative splicing of protein kinase CδII (PKCδII) and Bcl2 elevation 225.

A large set of MSC EV-associated miRNAs, including miR-22-3p, miR-25, miR-26, miR-31, miR-126, miR-138-5p, miR-146a-5p and miR-223-3p, were found to promote neuronal, astrocytic, oligodendrocytic and microglial survival by downregulating target genes including KDM6B, p53, KLF9, CH25H, TRAF6, ACVR2B, LCN2 or CysLT2R 185, 186, 188, 190, 197, 201-204, 209, 212, 213, 215, 216. Besides, miR-34c, miR-92b-3p and miR-361 transferred with EVs from normoxic or ischemic astrocytes increased neuronal survival by downregulating the targets TLR7 and cathepsin-B (CTSB) 205, 206, 217. Likewise, M2 microglial EV miR-124, miR-135a-5p and miR-137 increased neuronal survival and reduced astrocytic activation, proliferation and scar formation via mechanisms involving STAT3, thioredoxin interacting protein (TXNIP) and NOTCH1 downregulation 207, 210, 211. Neuroprotective effects were furthermore reported for miR-1290 derived from endothelial EVs 218. In the latter study, EV uptake by neurons occurred calveolin-1 dependently, and this uptake was increased by hypoxia-ischemia 218. Survival-promoting effects of patient serum-derived EV miR-124-3p and miR-126 via mechanisms involving DNA methylase-3b (DNMT3B) were noted in neurons and microglial cells 189, 208, whereas patient serum-derived miR-27-3p increased neuronal death via mechanisms involving PPARγ downregulation and microglial overactivation 219. The combined evidence of these studies demonstrates that various types of brain cells mutually influence responses to stroke via EV-associated ncRNAs.

A peculiar mechanism associated with ncRNA-induced neuroprotection appears to be the inhibition of autophagy in recipient cells. Autophagy is an evolutionarily conserved mechanism, which maintains cellular homeostasis by degrading misfolded or nonfunctional proteins or damaged organelles 226, 227. Upon severe cellular stress, excessive autophagy may result in cellular accumulation of toxic metabolites or cellular self‐degradation, ultimately resulting in cell death 228-230. Recent studies evaluating effects of MSC EVs showed that EV miR-25-3p protected primary neurons exposed to oxygen-glucose deprivation against injury via autophagy inhibition 202. On the molecular level, p53 expression was downregulated by miR-25-3p, resulting in the inhibition of BNIP3 activity and reduced autophagic flux examined by LC3-II levels. Application of a miR-25-3p oligonucleotide mimic promoted neuronal survival, whereas an miR-25-3p anti-oligonucleotide increased autophagic flux and cell death by mechanisms involving p53 overexpression and BNIP3 overactivation 202. Inhibitory effects on autophagy associated with neuronal survival were also described for circSHOC2 and miR-135a-5p released in astrocytic and M2 microglial EVs 200, 210. Hence, autophagy inhibition might represent a more general, hitherto underexplored mechanism via which EV-ncRNAs protect ischemic neurons.

### 4.2 Inflammation

Similar to the heart, proinflammatory cytokines, namely IL-1β, contribute to ischemic brain injury via pyroptosis. In the brain, proinflammatory cytokines, such as IL-6, TNF-α and IL-1β, are released from a variety of cells, including M1 microglial cells, astrocytes and neurons. ncRNAs transferred via EVs seem to regulate these inflammatory responses, as has been observed for EVs originating from a number of cell sources. Hence, miR-26b-5p, miR-126, miR-138-5p, miR-138-5p, miR-221-3, miR-223-3p and miR-542-3p released via MSC EVs inhibited inflammatory responses of neurons, microglial cells and astrocytes via CH25H, LCN2, RMRP, ATF3, CysLT2R and TLR4 downregulation, resulting in the inhibition of the TLR4, SMAD2, IRAK1/ TRAF6 and PI3K/ Akt/ mTOR pathways 186, 188, 190, 197, 212, 213, 216. Via this mechanism, neuronal survival was enhanced, microglial cells adopted a restorative M2 phenotype, and astrocytic inflammatory responses were inhibited. Likewise, miR-135a-5p derived from M2 microglial EVs reduced inflammatory responses of neurons via TXNIP downregulation, NLRP3 deactivation and decreased IL1β and IL18 formation 210, while neuronal EV miR-98 and miR-181c-3p promoted microglia survival and inhibited microglia phagocytosis or inhibited astrocyte inflammatory responses by PAFR or CXCL1 downregulation, respectively 192, 214. Patient serum-derived EV miR-124-3p inhibited microglial inflammatory responses by mechanisms involving ERK1/2, PI3K/ Akt and p38 MAPK deactivation 189, whereas patient serum-derived EV miR-27-3p promoted M1-like microglial activation via mechanisms involving PPARγ downregulation, resulting in increased cytokine formation and cell death 219.

### 4.3 Angiogenesis and neurogenesis

In models of cerebral ischemia, angiogenic effects have been shown for miR-181b and miR210 transferred via MSC EVs and for miR-126 transferred via endothelial EVs via mechanisms involving TRPM7 downregulation, HIF1α and VEGF elevation and TIMP3 reduction 187, 220, 221. When cultured under conditions of hypoxia, MSC EVs that were otherwise non-angiogenic adopted a recovery-promoting phenotype that reproducibly induced cerebral microvascular endothelial proliferation, migration and tube formation across a wide range of MSC sources 198. Compared with EVs from normoxic MSCs, hypoxic MSC EVs significantly increased miR-126-3p, miR-140-5p and let-7c-5p and reduced miR-186-5p, miR-370-3p and miR-409-3p in recipient endothelial cells 198. The delivery of these hypoxic MSC EVs *in vivo* to ischemic mice exposed to middle cerebral artery occlusion enhanced microvascular remodeling, increased microvascular densities, increased microvascular length and increased branching point densities, as revealed by 3D lightsheet fluorescence microscopy in the periinfarct rim 198. Newly formed microvessels act as guidance sheaths for neural progenitor cells migrating from progenitor cell niches to the stroke lesion. Delivery of miR-17-92, miR-26a and miR-124 shuttled via EVs from MSCs or urine-derived stem cells promoted post-ischemic neurogenesis via mechanisms involving histone deacetylase-6 (HDAC6) inhibition 222-224.

### 4.4 Neuronal plasticity

In response to stroke, axons and dendrites in the vicinity and at distance to the evolving brain infarct sprout, forming new synaptic connections 183. Cell-based therapeutics, including exogenously administered neural progenitor cells or MSCs, promote neuronal plasticity 231, 232. Within this process, EVs and their ncRNAs may play significant roles. Thus, the EV-derived lncRNA circSCMH1 was shown to increase dendritic length, dendritic branches and synaptic spines of ischemic cultured neurons in vitro and of periinfarct cortical neurons of rats exposed to photothrombotic stroke in vivo, as revealed by morphological Golgi-Cox staining analysis 191. EV circSCMH1 improved functional neurological recovery of ischemic rats, reduced microglial activation and reduced the formation of the proinflammatory cytokines IL1β, TNFα and IL6 191. The effect of circSCMH1 was mediated by binding methyl-CpG binding protein-2 (MeCP2), a nuclear transcription factor directly binding methylated DNA, as revealed by proteomic assays, RNA sequencing and transcriptional profiling studies 191. By MeCP2 binding, MeCP2 target gene transcription repression was released. In rat and mouse models of middle cerebral artery occlusion, neuronal plasticity and neurological recovery promoting effects were reported for MSC EV miR-17-92 and miR-133b and for endothelial cell EV miR-126 187, 195, 196, 222. Via mechanisms involving downregulation of PTEN, connective tissue growth factor (CTGF) or RhoA, the three miRNAs were found to increase axonal, dendritic and synaptic sprouting in the periinfarct tissue, as revealed by anterograde tract tracing analysis using biotinylated dextran amine combined with immunohistochemical stainings. In case of miR-17-92, the plasticity-promoting effects were associated with PI3K/ Akt/ mTOR activation and GSK3β deactivation 222. In case of miR-126, which was evaluated in a type-II diabetes stroke model, the neurorestorative effects were linked to a shift of macrophage polarization towards the M2 phenotype 187.

## 5. Overarching roles of EV-ncRNAs across pathophysiological conditions

lncRNAs, circRNAs, miRNAs and mRNAs form complex RNA networks that synergistically respond to stressors 233. As outlined in sections 2-4, several of these networks are highly active or inactive under conditions of hypoxia and ischemia, representing master regulators of gene expression. We are just starting to understand the complex biology behind these ncRNA networks. miRNAs recognize response elements on RNAs that mediate their interaction and binding. lncRNAs and circRNAs serve as competing endogenous RNAs to miRNAs, and thus act as miRNA sponges. mRNA binding of miRNA induces translational repression or instability, thus regulating protein translation 233. Importantly, lncRNAs do not only interact with miRNAs but can also target DNA transcription and mRNAs directly 234. Thus, EV-circSCMH1, which is decreased in plasma of stroke patients and periinfarct cortex of stroke mice, was found to induce post-ischemic dendritic and synaptic plasticity, antiinflammation and neurological recovery by binding the nuclear transcription factor MeCP2, resulting in release of MeCP2 mediated transcription repression 191 (see also section 4 and **Table 3**). Using antisense oligonucleotide studies and RNA immunoprecipitation assays on HT22 neuronal cells, lncRNA MALAT1, which is highly abundant in MSC EVs, was shown to promote neuronal survival and proliferation by mechanisms involving SRSF2 recruitment, alternative PKCδII splicing and Bcl2 elevation 225 (**Table 3**). For further insights into lncRNA-circRNA/ miRNA/ mRNA networks, the reader is referred to references 235-237. In view of their highly integrated mode of action, ncRNAs profoundly modify disease responses.

### 5.1 ncRNAs involved in more than one of three hypoxic conditions exhibit a large degree of overlaps regarding modes of action

From the above EV-ncRNA intervention studies, a total of 19 ncRNAs, including 3 lncRNAs and 16 miRNAs, have meanwhile been identified for which robust evidence suggests their involvement in more than one of the three hypoxic pathophysiological conditions. The modes of action of these ncRNAs have been summarized in **Table 4**. Notably, 8 of these 19 EV-ncRNAs have been shown to be involved in all three pathophysiological conditions. Including studies evaluating effects of ncRNAs irrelevant whether ncRNAs were associated with EVs, joint evidence in all three pathophysiological conditions exists for 16 of the 19 ncRNAs. Strikingly, the modes of action reveal a high degree of overlaps between the three pathophysiological states. Hence, studies describing promotion of cell survival, proliferation, migration or angiogenesis in one pathophysiological condition usually had corresponding actions in the two other conditions, as shown for lncRNA MALAT1, miR-21, miR-25, miR-31, miR-135, miR-146 and miR-210. Similarly, ncRNAs with roles in immune tolerance, antiinflammation or chemotherapy resistance in one condition also revealed related actions in the two other conditions, as shown for miR-98 and miR-223. Hence, corresponding actions in all three conditions have been reported for 9 of the 16 ncRNAs. Importantly, diverging actions have been reported for 3 ncRNAs. Perhaps due to the different nature of hypoxia, opposing actions were described for cancer compared to myocardial infarction and stroke in case of two ncRNAs. Hence, miR-133a, which was found to be abundant in cancer EVs at low concentration, reduced tumor cell proliferation, survival, migration and metastasis in gastric carcinoma and colorectal carcinoma 136, 238, whereas miR-133a-3p and miR-133b promoted cardiomyocyte survival and neuronal plasticity in myocardial infarction and stroke, respectively, and enhanced functional tissue recovery 162, 195, 196. Likewise, miR-328b-3p promoted tumor cell proliferation, migration, invasion and tumor growth in lung carcinoma 105, whereas miR-328b-3p augmented cardiomyocyte death and apoptosis in myocardial infarction 148 and augmented neuronal death and neuroinflammation in ischemic stroke 239. Besides, EV-associated miR-361 promoted tumor cell proliferation and survival in colorectal carcinoma 103 and neuronal survival in ischemic stroke 217, whereas cardiac-specific miR-361 overexpression reduced cardiomyocyte survival by increasing mitochondrial fission in myocardial infarction 240. Differences in the actions of EV-associated miR-361 and genetically overexpressed miR-361 may explain diverging findings in the two ischemia studies. Important for potential clinical translation, therapeutic miRNA modification may have opposite roles within the same disease category via distinct modes of actions. Hence, EV-associated miR-126 promoted tumor cell proliferation, angiogenesis and growth in hepatoblastoma and chronic myeloid leukemia via mechanisms involving CXCL12 and VCAM1 downregulation 129, 131, but inhibited tumor cell proliferation, colony formation, migration, invasion and survival in lung carcinoma via mechanisms including ITGA6 downregulation 130. NEAT1 overexpression promoted neuronal survival in ischemic stroke by regulating the MFN2/ SIRT3 pathway 241, whereas NEAT1 knockdown increased neuronal survival by inhibiting M1 microglia polarization via the Akt/ STAT3 pathway 242. Again in ischemic stroke, miR-494 agomir (that is, mimic) promoted neuronal survival, axonal plasticity and neurological recovery via HDAC3 downregulation 193, similar as miR-494 antagomir (that is, inhibitor), which increased neuronal survival and neurological recovery by reducing the Th1 helper cell shift and decreasing post-ischemic brain neutrophil infiltrates via HDAC2 upregulation 194, 243. In view of the multifaceted roles of ncRNAs, their therapeutic modulation is particularly prone to ambiguous actions in different types of cells. In case of therapeutic interventions, careful actions are needed in order to avoid contrary results of therapeutic interventions, i.e., therapeutic benefits in one cell type (e.g., in neuron) or via one pathway (e.g., MFN2/ SIRT3) vs. harmful actions in another cell type (e.g., brain invading T cells) or pathway (e.g., Akt/ STAT3). The genetic overexpression or knockdown and the delivery of miRNA agomirs or antagomirs are gross interventions that affect miRNA levels in a non-targeted way. In comparison, the delivery of miRNA-loaded EVs is more fine-tuned and allows targeting distinct types of cells.

## 6. EV-associated ncRNAs as therapeutic products or theranostic biomarkers

From studies in cancer, myocardial infarction and ischemic stroke, there is meanwhile solid evidence that EV-associated ncRNAs regulate gene responses in target tissues under conditions associated with hypoxia, modifying cell survival, proliferation, migration and differentiation and influencing disease outcomes in clinically relevant ways. In most pathophysiological conditions, we still lack of detailed information about the precise subcellular origin of therapeutically active EVs. Of note, the association of ncRNAs with EVs does not imply that ncRNAs are exosome constituents 15, and concerns have been raised whether ncRNAs are released from cells as part of the EVs 17. EVs are widely isolated by differential ultracentrifugation 86, which enriches non-EV constituents including lipoproteins 87 that contain large amounts of ncRNAs 88. Bead-capturing experiments revealed that EVs captured by GM1 binding cholera toxin b were largely devoid of RNAs (see also section 1.5). EV contents may differ depending on pathophysiological conditions. Besides exosomes, microvesicles might contain ncRNAs. Irrespective of the precise ncRNA and EV origin and overriding open methodological questions, intervention studies in models of cancer, myocardial infarction and stroke consistently revealed therapeutic actions of EV preparations that were associated with ncRNAs. The observation raises the question about the utility of EV-associated ncRNAs as therapeutics or theranostics in human patients.

### 6.1 ncRNA-loaded EVs as therapeutic products

Representing instable single-strand RNA molecules, miRNAs are rapidly degraded in the blood by RNAses, unless specifically protected. EVs are abundant in virtually all body fluids, protecting ncRNAs from degradation. Representing nanoparticles covered by lipid bilayer membrane, EVs are capable of transmitting complex biological information to defined target cells. The presence of multiple signals in a single EV allows inducing synergistic cellular responses. When evaluating EV actions, we must consider that not all EVs transmit biological information and that some EVs eliminate waste products, including RNAs, from hypoxic cells (see section 1.1). The proper definition of cells or tissues of origin is decisive in the development of EV products 244. In the development of EV-based therapeutics, we have to be aware that EV contents, including ncRNAs, may greatly differ between EV preparations, even when these preparations are performed from the same source cells 198, 245. This raises the need of potency assays evaluating the efficacy of each EV preparation before this individual preparation is administered to human patients 246. When considering ncRNAs as therapeutic EV contents, it must be taken into account that the biological effect of a given ncRNA may differ between pathophysiological settings and disease-relevant target cell types. As example, miR-494 was shown to promote post-ischemic neuronal survival, axonal plasticity and neurological recovery via its target HDAC3 in one setting 193, while it had opposite effects by modulating Th1 helper cell shifts and brain neutrophil infiltrates via HDAC2 in two other studies 194, 243 (see also **Table 4**). Thorough studies in animal models would be required elucidating various modes of action before clinical proof-of-concept studies are performed. Considering their biological properties that resemble cellular therapeutics, but are more easy to handle and lack intrinsic risks of cellular therapeutics (such as malignant transformation) 244, the administration of EVs is an elegant strategy to boost hypoxic tissues. EVs might potentially be loaded with ncRNAs that prevent disease progression or improve disease outcome, or be loaded with inhibitors or siRNAs for ncRNAs that promote disease progression or deteriorate disease outcome. EV ncRNA contents might possibly be modified by transgenic techniques. Following transfection of cancer cells with plasmid DNA encoding for wild-type p53 and miR-125b, Trivedi et al. observed that the ncRNA profile of EVs was altered, shifting the polarization of recipient macrophages towards the M1 phenotype 247.

### 6.2 EV-ncRNAs as theranostic biomarkers

EVs carry surface markers specifying their cellular origin and their source cell's activation state. Cell type by cell type, tissue responses can be tracked in remote body fluids. In multiple dimensions, detailed information can be obtained about hypoxic or ischemic tissue states. For this type of biomarker analysis, the term liquid biopsy has been coined. Deregulated EV-ncRNA levels may be used as diagnostic or prognostic markers. As such, Que et al. reported that EV miR-17-5p and miR-21 were elevated 3.2-fold and 5.9-fold, respectively, in the serum of patients with pancreatic adenocarcinoma, and serum miR-21 furthermore differentiated pancreatic adenocarcinoma from chronic pancreatitis 248. In myocardial infarction, serum EV-derived miR-1915-3p, miR-457, and miR-3656 were significantly less abundant compared with patients with stable coronary artery disease, and the miR-3656 level was positively correlated with left ventricular ejection fraction 249. Indeed, by comparing EV-ncRNAs abundancies at baseline, before and after treatment with subsequent correlation with clinical variables, such biomarkers offer an elegant possibility to predict disease outcomes and therapy responses. For instance, in pancreatic adenocarcinoma, the level of serum EV circPDE8A was positively correlated with lymphatic invasion, TNM stage and poor survival rate 250. On the contrary, miR‐134 - known as a brain‐specific miRNA - has been associated with neuronal injury under conditions of ischemic stroke 251. Abundances of EV-derived miR‐134 were significantly increased in stroke patients, where they were positively correlated with neurological deficits, stroke volume and functional outcome 252. A detailed and comprehensive analysis of the utility of miRNAs as diagnostic markers is beyond the scope of this review. For more details, the reader is referred to Table 5 102, 108, 110, 113, 114, 248-250, 252-266. Specifically, the here-presented data on the role of EV-ncRNAs in the development of chemotherapy resistance (summarized in section 2.4) supports their role in treatment monitoring. In a variety of cancers, including pancreatic carcinoma, colorectal carcinoma, ovarian carcinoma, hepatocellular carcinoma and lung carcinoma, pathophysiologically grounded evidence was collected supporting a role of defined ncRNAs in chemotherapy resistance development (summarized in section 2.4 and **Figure 2**). With the identification of concentration cutoffs, these EV-ncRNAs could now be used for chemotherapy monitoring. As theranostics, ncRNA-EVs should provide clinically significant information, whether a given drug is still likely to have preserved its actions or whether it should be exchanged due to drug resistance development. For example, hypoxia-associated miR-21 abundance has revealed its utility as theranostic marker in non-small cell lung carcinoma, where high miR-21 abundance was associated with short survival in patients receiving chemotherapy with cisplatin, but not in patients not receiving chemotherapy 94. The predictive value of EV-ncRNAs may further be enhanced by evaluating EV-ncRNA combinations or EV-ncRNA combinations with classical biomarkers. For instance, serum α-fetoprotein was found to have an insufficient sensitivity and specificity in hepatocellular carcinoma patients, resulting in an unacceptably high false-negative detection rate 267. Combining α-fetoprotein with serum EV ENSG00000258332.1 and LINC00635 markedly increased α-fetoprotein's sensitivity and specificity. With the new biomarker, a higher percentage of patients could now be classified correctly 267.

## 7. Conclusion and perspectives

We herein showed that cancer, myocardial infarction and ischemic stroke, which represent the most prevalent medical conditions resulting in disability and death 3, profoundly regulate EV-associated ncRNAs, controlling physiological responses including cell survival and proliferation, migration, drug resistance, angiogenesis and neuronal plasticity. As underlying mechanism, hypoxia, which decisively influences tissue fate in all three conditions 1, 2, was found to control endogenous ncRNA responses, some ncRNAs being upregulated and others downregulated upon hypoxia. In several studies, the delivery of ncRNA-loaded EVs harvested from healthy human blood, perilesional tissue, progenitor cells (including MSCs) or antiinflammatory immune cell subsets (such as M2 microglia) allowed attenuating disease progression and restoring functional tissue recovery. Remarkably, EV-ncRNAs exhibited a large degree of overlaps regarding their modes of action across pathophysiological states. Thus, EV-ncRNAs open fascinating perspectives as theranostic biomarkers and besides this, but more challenging, perhaps also as therapeutic products. With respect to clinical translation, major challenges remain with respect to the large-scale isolation and preparation of EVs, the reproducibility of EV preparations under defined culturing conditions as well as the development of potency assays, which ensure that EV activity characteristics are maintained in a given EV preparation before this individual preparation is administered to human patients 245, 246, 268. Only with such skills, we can ensure the success of clinical proof-of-concept trials. Currently, there are 23 clinical trials on EV-enveloped ncRNAs registered at https://clinicaltrials.gov/ in diverse medical conditions. Among these studies, currently eight trials are active and recruiting, four are active but not recruiting, four are not recruiting and seven are completed. The results of these ongoing studies are envisaged with great interest. Meanwhile, ongoing preclinical efforts will sharpen our understanding of the clinical potential of EV-ncRNA technologies.

## Figures and Tables

**Table 1:** Preclinical studies assessing the effect of ncRNAs transferred via EVs in the hypoxic tumor microenvironment.

Authors [reference]	Cancer type	ncRNAs	EV provenance	Recipient cell	Primary action	Mechanism of action
**Xue et al.** 102	Bladder carcinoma	lncR UCA1	5637 cancer cells	UMUC2 cancer cells	Tumor cell proliferation	Promotion of epithelial-mesenchymal transition
**Zhang et al.** 123	Lung carcinoma	lncR MALAT1	Patient serum	A549 and H1299 cancer cells	Tumor cell proliferation, migration and survival	Not determined
**Rong et al.** 124	Lung carcinoma	lncR MALAT1	Patient serum, A549 and H1299 cancer cells	A549 and H1299 cancer cells	Tumor cell proliferation, invasion and survival	miR-515 sponging, EEF2 upregulation,
**Wang et al.** 125	Lung carcinoma	lncR MALAT1	A549 and H1299 cancer cells	A549 and H1299 cancer cells	Tumor cell proliferation, colony formation and glycolysis	miR-613 sponging, COMMD8 upregulation
**Zhou et al.** 122	Breast carcinoma	lncR NEAT1	Patient serum	MCF-7 and MDA-MB-231 cancer cells	Tumor cell proliferation, migration, invasion and metastasis	miR-141-3p sponging, KLF12 upregulation
**Takahashi et al.** 120	Hepatocellular carcinoma	lncR ROR	HepG2 cancer cells	HepG2 cancer cells	Tumor cell proliferation	miR-145 downregulation, HIF1α stabilization
**Chen et al.** 95	Ovarian carcinoma	miR-21-3p/ miR-125 b-5p/ miR-181d-5p	SKOV3 cancer cells	SKOV3 cancer cells/ macrophages	Tumor cell proliferation/ immune tolerance	HIF-1α/ HIF-2α stabilization, M2 macrophage polarization
**Hu et al.** 129	Hepatoblastoma	miR-126	huH6 and HepG2 cancer cells	huH6 and HepG2 cancer cells, MSCs	Tumor cell proliferation, tumor growth	MSC differentiation into cancer cells
**Katakowski et al.** 128	Glioma	miR-146b	MSCs	Glioma cells	Tumor cell proliferation/ tumor growth	Not determined
**Meng et al.** 104	Renal carcinoma	miR-155	786-O and Caki-1 cancer cells	786-O and Caki-1 cancer cells	Tumor cell proliferation	FOXO3 downregulation
**Liu et al.** 105	Lung carcinoma	miR-328-3p	MSCs	A549 and H125 cancer cells	Tumor cell proliferation, migration, invasion/ tumor growth	Promotion of epithelial-mesanchymal transition; NF2 downregulation, inhibition of Hippo pathway activation
**Li et al.** 103	Colorectal carcinoma	miR-361-3p	CRC cancer cells	HCT116 and HT29 cancer cells	Tumor cell proliferation, survival; tumor growth	TRAF3 downregulation, NF-κB activation
**Yu et al.** 98	Hepatocellular carcinoma	miR-1273f	Huh7 and 97H cancer cells	Huh7 and 97H cancer cells	Tumor cell proliferation	LHX6 downregulation
**Yang et al.** 136	Colorectal carcinoma	circ-133	Patient serum, SW 480 and HCT 116 cancer cells	SW 480 and HCT 116 cancer cells	Tumor cell migration/ metastasis	miR-133a sponging, GEF-H1 and RhoA elevation
**Li et al.** 114	Oral squamous cell carcinoma	miR-21	Patient serum	SCC-9 and CAL-27 cancer cells	Tumor cell migration/ invasion	HIF1α/HIF2α stabilization
**Liu et al.** 133	Hepatocellular carcinoma	miR-25-5p	HuH-7 and HCCLM3 c cancer ells	HuH-7 and HCCLM3 cancer cells	Tumor cell migration/ invasion	Not determined
**Yu et al.** 121	Lung carcinoma	miR-31-5p	A549 and H1299 cancer cells	A549 and H1299 cancer cells	Tumor cell migration/ invasion	SATB2-revered epithelial-mesenchymal transition, ERK1/2 activation
**Zhang et al.** 97	Lung carcinoma	miR-193a-3p/ miR-210-3p/ miR-5100	MSCs	A549, H358, H460 and LLC cancer cells	Tumor cell migration/ invasion	STAT3 activation, epithelial-mesenchymal transition
**Li et al.** 130	Lung carcinoma	miR-126	Patient serum	A549 and H460 cancer cells	Inhibition of tumor cell proliferation, colony formation, migration, invasion and survival	ITGA6 downregulation
**Guo et al.** 106	Pancreatic carcinoma	lncR UCA1	MIA PaCa-2 cancer cells	Endothelial cells	Angiogenesis	miR-96-5p sponging, AMOTL2 repression reversal
**Hsu et al.** 108	Lung carcinoma	miR-23a	CL1-5 cancer cells	Endothelial cells	Angiogenesis	PHD1/ PHD2 downregulation, HIF1α stabilization
**Zeng et al.** 134	Colorectal carcinoma	miR-25-3p	Patient serum and CRC cancer cells	Endothelial cells	Angiogenesis, vascular permeability, metastasis	KLF2 and KLF4 downregulation
**Taverna et al.** 131	Chronic myeloid leukemia	miR-126	LAMA84 cancer cells	Endothelial cells	Angiogenesis	CXCL12 and VCAM1 downregulation
**Umezu et al.** 101	Multiple myeloma	miR-135b	RPMI8226 cancer cells	Endothelial cells	Angiogenesis	FIH downregulation, HIF1α stabilization
**Tadokoro et al.** 107	Leukemia	miR-210	K562 cancer cells	Endothelial cells	Angiogenesis	EFNA3 downregulation
**Mao et al.** 109	Lung carcinoma	miR-494	A549 cancer cells	Endothelial cells	Angiogenesis, tumor growth	PTEN downregulation, Akt/ eNOS activation
**Li et al.** 119	Oral squamous cell carcinoma	miR-21	Cal-27 and SCC9 cancer cells	γδ T cells	Immune tolerance	γδ T cell deactivation through PTEN/PD-L1 axis regulation
**Berchem et al.** 111	Different cancers, including lung carcinoma	miR-23a	IGR-Heu and K562 cancer cells	NK cells	Immune tolerance	NK cell deactivation through CD107a downregulation
**Ye et al.** 110	Nasopharyngeal carcinoma	miR-24-3p	TW03, C666 and CNE2 cancer cells	T cells	Immune tolerance	T cell deactivation through FGF11 downregulation, ERK1/2 and STAT1/3 activation and STAT5 deactivation
**Guo et al.** 93	Glioma	miR-10a/ miR-21	P3 and GL261 cancer cells	MDSCs	Immune tolerance	MDSC expansion, RORA and PTEN downregulation
**Yang et al.** 126	Melanoma	lncR NEAT1	MSCs	Macrophages	Immune tolerance	M2 macrophage polarization through miR-374 sponging, LGR4-dependent IQGAP1 upregulation
**Hsu et al.** 116	Lung carcinoma	miR-103a	CL1-5 cancer cells	Macrophages	Immune tolerance	M2 macrophage polarization through PTEN downregulation, Akt/ STAT3 activation
**Wang et al.** 117	Pancreatic carcinoma	miR-301a	PANC-1 cancer cells	Macrophages	Immune tolerance	M2 macrophage polarization through PTEN downregulation, PI3Kγ activation
**Chen et al.** 118	Ovarian carcinoma	miR-940	SKOV3 cancer cells	Macrophages	Immune tolerance	M2 macrophage polarization
**Park et al.** 100	Melanoma	let-7a miR	B16 melanoma cells	Macrophages	Immune tolerance	M2 macrophage polarization through inhibition of insulin/ Akt/ mTOR signaling
**Yang et al.** 127	Glioma	lncR MALAT1	Glioma stem cells	Microglia	Proinflammatory response	miR-129-5p sponging, HMGB1 upregulation, IL6, IL8 and TNFα release in response to lipopolysaccharide exposure increased
**Zeng et al.** 112	Pancreatic carcinoma	circZNF91	BxPC-3 and SW1990 cancer cells	BxPC-3 cancer cells	Chemotherapy resistance	miR-23b-3p sponging, SIRT1 upregulation, HIF1α stabilization
**Wang et al.** 135	Colorectal carcinoma	ciRS-122	SW480 and L‐OHP cancer cells	SW480 cancer cells	Chemotherapy resistance, glycolysis promotion	miR-122 sponging, PKM2 upregulation
**Takahashi et al.** 99	Hepatocellular carcinoma	lncR ROR	HepG2 cancer cells	HepG2 cancer cells	Chemotherapy resistance	CD133+ cell formation
**Dong et al.** 94	Lung carcinoma	miR-21	A549 cancer cells	A549 cancer cells	Chemotherapy resistance	PTEN downregulation
**Guo et al.** 132	Ovarian carcinoma	miR-98-5p	Cancer-associated fibroblasts	A2780 cancer cells	Chemotherapy resistance	CDKN1A downregulation
**Zhu et al.** 113	Ovarian carcinoma	miR-223	Macrophages	SKOV3 cancer cells	Chemotherapy resistance	PTEN downregulation, PI3K/Akt activation
**Chen et al.** 115	Oral squamous cell carcinoma	miR-340-5p	Te13, Te1 and Eca109 cancer cells	Te13, Te1 and Eca109 cancer cells	Radiotherapy resistance	KLF1 downregulation

**Abbreviations:** EVs, extracellular vesicles; lncR, long non-coding RNA; MDSCs, myeloid-derived suppressor cells; MSCs, mesenchymal stromal cells.

**Table 2:** Preclinical studies assessing the effects of ncRNAs transferred via EVs in models of myocardial infarction.

Authors [reference]	ncRNAs	EV provenance	Recipient cell	Ischemia model	Primary action	Mechanism of action
**Diao et al.** 153	lncR UCA1	MSCs	Cardiomyocytes	H/R, LAD ligation	Cell survival, autophagy inhibition	miR-143 sponging/ Bcl2 elevation
**Shyu et al.** 154	lncR MALAT1	Cardiomyocytes	Cardiomyocytes, endothelial cells	H/R, LAD ligation	Cell survival	miR-92a sponging, KLF2 and CD31 elevation
**Kenneweg et al.** 149	lncR NEAT1	Cardiomyocytes	Cardiomyocytes, fibroblasts	LAD ligation	Cell survival, cardiac function recovery, antifibrosis	NEAT1 transcriptionally upregulated in large EVs by hypoxia through HIF2α
**Chen et al.** 155	lncR KLF3-AS1	Cardiomyocytes	Cardiomyocytes, BMSCs	LAD ligation	Cell survival	miR-23c sponging, STAT5B upregulation
**Mao et al.** 156	lncR KLF3-AS1	MSCs	Cardiomyocytes	H/R, LAD ligation	Cell survival, Pyroptosis inhibition	miR-138-5p sponging, SIRT1 upregulation
**Li et al.** 140	lncR HCP5	MSCs	Cardiomyocytes	H/R, LAD ligation	Cell survival	miR-497 sponging, IGF1 upregulation, PI3K/Akt activation
**Gu et al.** 141	miR-21	Patient serum	Cardiomyocytes	OGD/R, LAD ligation	Cell survival	PDCD4 downregulation
**Song et al.** 157	miR-21	HEK293T	Cardiomyocytes, endothelial cells	H2O2, LAD ligation	Cell survival	PDCD4 downregulation
**Zhang et al.** 147	miR-24	MSCs	Cardiomyocytes	LAD ligation	Cell survival, cardiac function recovery	Bax, caspase-3 and activated caspase-3 reduction
**Peng et al.** 181	miR-25	MSCs	Cardiomyocytes	OGD/R, LAD ligation	Cell survival	FASL and PTEN downregulation, EZH2 and H3K27me3 reduction elevating eNOS and SOCS3
**Pu et al.** 142	miR-30e	MSCs	Cardiomyocytes	H/R, LAD ligation	Cell survival, cardiac function recovery	LOX1 downregulation, NF-κB p65 and caspase-9 deactivation
**Zhang et al.** 143	miR-98-5p	MSCs	Cardiomyocytes	LAD ligation	Cell survival, cardiac function recovery, antiinflammation	TLR4 downregulation, PI3K/ Akt activation, reduced macrophage infiltration
**Zhu et al.** 159	miR-125b	MSCs	Cardiomyocytes	H/R, LAD ligation	Cell survival, cardiac function recovery	p53/BAK1 downregulation
**Chen et al.** 176	miR-126	Endothelial cells	Cardiomyocytes	MCAO	Cell survival, cardiac function recovery	Vascular cell adhesion protein-1 and monocyte chemotactic protein-1 reduction
**Luo et al.** 160	miR-126	MSCs	Cardiomyocytes, endothelial cells	LAD ligation	Cell survival, antiinflammation, antifibrosis, angiogenesis	Reduced proinflammatory cytokine formation
**Zheng et al.** 158	miR-129	Endothelial cells	Cardiomyocytes	OGD/R, LAD ligation	Cell survival, antiinflammation	TLR4 downregulation, NF-κB and NLRP3 inflammasome deactivation
**Wang et al.** 161	miR‐129-5p	MSCs	Cardiomyocytes	Coronary artery ligation	Cell survival, antiinflammation, antifibrosis	HMGB1 downregulation
**Zhu et al.** 162	miR-133a-3p	MSCs	Cardiomyocytes	H/SD, LAD ligation	Cell survival, cardiac function recovery	Akt activation
**Pan et al.** 163	miR‐146a	MSCs	Cardiomyocytes	H/R, LAD ligation	Cell survival, antiinflammation, antifibrosis	EGR1 downregulation, TLR4/ NFκB deactivation
**Wu et al.** 144	miR-150-5p	MSCs	Cardiomyocytes	LAD ligation	Cell survival, cardiac function recovery	Bax downregulation
**Li et al.** 145	miR-185	MSCs	Cardiomyocytes	Coronary artery ligation	Cell survival	SOCS2 downregulation
**Barile et al.** 177	miR-210	Cardiac progenitor cells	Cardiomyocytes	LAD ligation	Cell survival	Ephrin-A3 and PTP1b downregulation
**Cheng et al.** 178	miR-210	MSCs	Cardiomyocytes	LAD ligation	Cell survival	AIFM3 downregulation
**Wu et al.** 146	miR-212-5p	MSCs	Cardiomyocytes	LAD ligation	Cell survival, antifibrosis	NLRC5 downregulation, VEGF/ TGFβ1/ SMAD deactivation
**Ke at al.** 164	miR-218-5p/ miR-363-3p	Endothelial progenitor cells	Cardiomyocytes	LAD ligation	Cell survival, antifibrosis, angiogenesis	p53/ JMY downregulation
**Fu et al.** 165	miR-338	MSCs	Cardiomyocytes	H_2_O_2_, LAD ligation	Cell survival, cardiac function recovery	MAP3K downregulation, JNK/Bax reduction, Bcl2 elevation
**Wang et al.** 150	miR-671	ASCs	Cardiomyocytes	OGD/R, LAD ligation	Cell survival, antiinflammation, antifibrosis	TGFBR2 downregulation, SMAD2 deactivation
**Sanchez-Sanchez et al.** 166	miR-4732-3p	MSCs	Cardiomyocytes, endothelial cells	OGD/R, LAD ligation	Cell survival, cardiac function recovery, antifibrosis, angiogenesis	Not determined
**Lin et al.** 167	lncR HCG15	Patient serum	Cardiomyocytes	H/R, LAD ligation	Cell death/ apoptosis, proinflammation	NF-κB/ p65 and p38 activation, IL1, IL6 and TNFα upregulation
**Ning et al.** 168	miR-153-3p	MSCs	Cardiomyocytes, endothelial cells	OGD/R	Cell death	ANGPT1 downregulation, VEGF/ VEGFR2/ PI3K/ Akt/ eNOS deactivation
**Huang et al.** 148	miR-328-3p	Cardiomyocytes	Cardiomyocytes	LAD ligation	Cell death/ apoptosis	Caspase-3 activation
**He et al.** 169	miR-21-5p	Patient serum	Endothelial cells	Orthotopic xenograft model	Angiogenesis, vascular permeability	KRIT1 downregulation, β-catenin activation, VEGF/ CCND1 elevation
**Zhu et al.** 170	miR-31	MSCs	Endothelial cells	HLI, LAD ligation	Angiogenesis, cardiac function recovery	FIH1 downregulation, HIF1α elevation
**Wang et al.** 179	miR-210	MSCs	Endothelial cells	LAD ligation	Angiogenesis, cardiac function recovery	EFNA3 downregulation
**Yang et al.** 180	miR-223	MSCs	Endothelial cells	H_2_O_2_, LAD ligation	Angiogenesis, antiinflammation, antifibrosis	P53 downregulation, S100A9 reduction
**Youn et al.** 171	miR-322	Cardiac progenitor cells	Endothelial cells	LAD ligation	Angiogenesis	NOX2 and reactive oxygen species (ROS) elevation
**Li et al.** 172	miR-486-5p	MSCs	Endothelial cells, cardiomyocytes	LAD ligation	Angiogenesis, cardiac function recovery	MMP19 downregulation, VEGFA elevation due to reduced cleavage
**Liu et al.** 173	miR‑494‑3p	Dendritic cells	Endothelial cells	H/R, LCA ligation	Angiogenesis	VEGF elevation
**Climent et al.** 174	miR-143/ miR-145	Smooth muscle cells	Endothelial cells	NA	Inhibition of angiogenesis, inhibition of endothelial proliferation	HKII and integrin-β8 downregulation, respectively
**Jiang et al.** 175	miR-2p8b	Patient plasma	Endothelial cells	NA	Inhibition of angiogenesis, promotion of endothelial death	CDKN1A, FAK, RAF1, MAPK1 and Bax upregulation, Bcl2 downregulation

**Abbreviations:** EVs, extracellular vesicles; lncR, long non-coding RNA; MSC, mesenchymal stromal cells; H/R: hypoxia-reoxygenation; LAD, left anterior descending artery; OGD/R, oxygen-glucose deprivation and reoxygenation/ recultivation; MCAO, middle cerebral artery occlusion; H/SD, hypoxia and serum deprivation; HLI, hindlimb ischemia; LCA, left coronary artery; NA, not available.

**Table 3:** Preclinical studies assessing the effects of ncRNAs transferred via EVs in ischemic stroke models.

Authors [reference]	ncRNAs	EV provenance	Recipient cell	Ischemia model	Primary action	Mechanism of action
**Chen et al.** 200	circSHOC2	Primary astrocytes	Primary neurons	OGD, MCAO	Cell survival, autophagy inhibition	miR-7670-3p sponging/ SIRT1 elevation
**El Bassit et al.** 225	lncR MALAT1	MSCs	HT22 neuronal cells	Oxidative stress	Cell survival and proliferation	SRSF2 recruitment, alternative PKCδII splicing, Bcl2 elevation
**Zhang et al.** 201	miR-22-3p	MSCs	Primary neurons	OGD, MCAO	Cell survival	KDM6B downregulation, BMP2/ BMF deactivation
**Kuang et al.** 202	miR-25	MSCs	Primary neurons	OGD, MCAO	Cell survival, autophagy inhibition	p53 downregulation, BNIP3 deactivation, reduced LC3-II abundance
**Hou et al.** 203	miR-26a	MSCs	Primary neurons	OGD, MCAO	Cell survival	KLF9 downregulation, TRAF2 and KLF2 elevation
**Li et al.** 197	miR-26b-5p	MSCs	SH-SY5Y, PC12, primary microglia	OGD, MCAO	Cell survival, antiinflammation	CH25H downregulation, TLR4 deactivation, inhibition of M1 microglia polarization
**Lv et al.** 204	miR-31	MSCs	Primary neurons	OGD, MCAO	Cell survival, functional neurological recovery	TRAF6 downregulation, IRF5 elevation, Bax/ activated caspase-3 reduction
**Wu et al.** 205	miR-34c	Astrocytes	N2a neuronal cells	OGD, MCAO	Cell survival	TLR7 downregulation, NFκB/MAPK deactivation
**Xu et al.** 206	miR-92b-3p	Primary astrocytes	Primary neurons	OGD	Cell survival	Not determined
**Yang et al.** 192	miR-98	Primary neurons	Primary microglia	OGD, MCAO	Cell survival, antiinflammation	PAFR downregulation, inhibition of microglia phagocytosis
**Li et al.** 207	miR-124	M2 BV2 microglia	Primary astrocytes	OGD, MCAO	Cell survival, inhibition astrocytic activation, proliferation and scar formation, functional neurological recovery, antiinflammation	STAT3 downregulation, GFAP reduction, nestin elevation
**Qi et al.** 189	miR-124-3p	Patient serum	BV2 microglia	AIS patients	Cell survival, antiinflammation	ERK1/2, PI3K/ Akt and p38 MAPK deactivation
**Cui et al.** 208	miR-126	Patient serum	SH-SY5Y neuronal cells	RIPC	Cell survival	DNMT3B downregulation
**Geng et al.** 190	miR-126	MSCs	Neurons, endothelial cells, BV2 microglia	OGD, MCAO	Cell survival, functional neurological recovery, anti-inflammation, neurogenesis, angiogenesis	Reduced microglial activation
**Feng et al.** 185	miR-132	MSCs	Primary neurons	OGD, MCAO	Cell survival	ACVR2B downregulation, SMAD2/ c-Jun inhibition
**Xiao et al.** 209	miR-134	MSCs	Primary oligodendrocytes	OGD	Cell survival	Caspase-8 deactivation
**Liu et al.** 210	miR-135a-5p	M2 microglia	HT-22 neuronal cells	OGD, MCAO	Cell survival, antiinflammation, autophagy inhibition	TXNIP downregulation, NLRP3 deactivation, reduced IL1β and IL18 formation
**Zhang et al.** 211	miR-137	M2 microglia	Primary neurons	OGD, MCAO	Cell survival, functional neurological recovery	NOTCH1 downregulation
**Deng et al.** 212	miR-138-5p	MSCs	Primary astrocytes	OGD, MCAO	Cell survival, antiinflammation	LCN2 downregulation, IL1β, IL6 and TNFα reduction, Bcl2 elevation, Bax reduction
**Zhang et al.** 213	miR-146a-5p	MSCs	BV2 microglia	OGD, MCAO	Cell survival, antiinflammation, functional neurological recovery	IRAK1/ TRAF6 deactivation, reduced microglial activation
**Song et al.** 214	miR-181c-3p	Primary neurons	Primary astrocytes	OGD, MCAO	Cell survival, antiinflammation	CXCL1 downregulation, reduced astrocyte activation
**Zhong et al.** 215	miR-206/ miR-1-3p	MSCs	Primary neurons	OGD	Cell survival	RMRP downregulation, PI3K/ Akt/ mTOR deactivation, eNOS elevation
**Ai et al.** 186	miR-221-3p	MSCs	Primary neurons	OGD, MCAO	Cell survival, antiinflammation	ATF3 downregulation
**Zhao et al.** 216	miR-223-3p	MSCs	BV2 microglia	OGD, MCAO	Cell survival, antiinflammation, functional neurological recovery	CysLT2R downregulation, M2 microglia polarization
**Bu et al.** 217	miR-361	Primary astrocytes	PC12 neuronal cells	OGD, MCAO	Cell survival	CTSB downregulation, AMPK/ mTOR deactivation
**Cai et al.** 188	miR-542-3p	MSCs	HA1800 astrocytes	OGD, MCAO	Cell survival, antiinflammation	TLR4 downregulation, ROS, IL6, TNFα and MCP1 reduction
**Yue et al.** 218	miR-1290	Endothelial cells	Primary neurons	OGD, MCAO	Cell survival	Neuronal EV uptake caveolin-1 dependent, increased by hypoxia-ischemia
**Ye et al.** 219	miR-27-3p	Patient serum	BV2 microglia	MCAO	Cell death, inflammation, compromised neurological recovery	PPARγ downregulation, microglial overactivation, proinflammatory cytokine formation
**Yang et al.** 220	miR-181b	MSCs	Brain microvascular endothelial cells	OGD, MCAO	Angiogenesis	TRPM7 downregulation, HIF1α and VEGF elevation, TIMP3 reduction
**Zhang et al.** 221	miR-210	MSCs	Brain microvascular endothelial cells	MCAO	Angiogenesis	Integrin-β3, VEGF and CD34 elevation
**Gregorius et al.** 198	-	MSCs	Brain microvascular endothelial cells	OGD, MCAO	Angiogenesis	Hypoxic MSC preconditioning induces angiogenic activity. miR-126-3p, miR-140-5p, let-7c-5p upregulated, miR-186-5p, miR-370-3p, miR-409-3p downregulated in endothelial cells in response to hypoxic but not normoxic MSC EVs
**Ling et al.** 223	miR-26a	Urine-derived stem cells	Neural stem cells	OGD, MCAO	Neurogenesis	HDAC6 inhibition
**Yang et al.** 224	miR-124	MSCs	Neural progenitor cells	Focal cortical ischemia	Neurogenesis	Not determined
**Yang et al.** 191	circSCMH1	Genetically engineered HEK293T cells	Neurons, glial cells, leukocytes	Photothrombosis	Neuronal (=dendritic and synaptic) plasticity, functional neurological recovery, antiinflammation	Release of MeCP2 transcription repression, microglial activation reduced, IL1β, TNFα and IL6 formation reduced
**Xin et al.** 222	miR-17-92	MSCs	Neurons, glial cells	MCAO	Neuronal (=axonal, dendritic and synaptic) plasticity, neurogenesis, functional neurological recovery, myelin remodeling	PTEN downregulation, PI3K/ Akt/ mTOR activation, GSK3β deactivation
**Venkat et al.** 187	miR-126	Endothelial cells	Neurons, endothelial cells, oligodendrocytes, microglia	Photothrombosis	Neuronal (=axonal) plasticity, functional neurological recovery, myelin remodeling, angiogenesis	M2 macrophage polarization
**Xin et al.** 195, 196	miR-133b	MSCs	Neurons, astrocytes	MCAO	Neuronal (=axonal) plasticity, functional neurological recovery	CTGF and RhoA downregulation

**Abbreviations:** EVs, extracellular vesicles; MSCs, mesenchymal stromal cells; OGD, oxygen-glucose deprivation; MCAO, middle cerebral artery occlusion; AIS, acute ischemic stroke; RIPC, remote ischemic preconditioning.

**Table 4:** ncRNAs transferred via EVs that have been reported to participate in more than one of the three hypoxic conditions.

ncRNAs	Modes of action in cancer	Modes of action in myocardial infarction	Modes of action in ischemic stroke
**lncR UCA1**	Promotes tumor cell proliferation and tumor-associated angiogenesis 102, 106	Promotes cardiomyocyte survival and inhibits autophagy 153	Not assessed
**lncR MALAT1**	Promotes tumor cell proliferation, migration, invasion, colony formation and glycolysis; promotes inflammation 123-125, 127	Promotes cardiomyocyte and endothelial cell survival 154	Promotes neuronal survival 225
**lncR NEAT1**	Promotes tumor cell proliferation, migration, invasion, metastasis, immune tolerance and chemoresistance 122, 126, 269	Promotes cardiomyocyte and fibroblast survival; inhibits fibrosis 149	[NEAT1 overexpression promotes neuronal survival via MFN2/ SIRT3 pathway 241; NEAT1 knockdown promotes neuronal survival by inhibiting M1 microglia polarization via Akt/ STAT3 pathway 242
**miR-21**	Promotes tumor cell proliferation, migration and invasion; induces immune tolerance via M2 macrophage polarization, γδ T cell deactivation and myeloid-derived suppressor cell expansion; induces chemoresistance 93-95, 114, 119	Promotes cardiomyocyte and endothelial cell survival; promotes periinfarct angiogenesis and vascular permeability 141, 157, 169	[miR-21 agomir promotes neuronal survival 270
**miR-24**	Induces immune tolerance by T cell deactivation 110	Promotes cardiomyocyte survival and cardiac function recovery 147	[miR-24 agomir promotes neuronal survival 271
**miR-25**	Promotes tumor cell migration, invasion, angiogenesis and metastasis 133, 134	Promotes cardiomyocyte survival 181	Promotes neuronal survival and inhibits autophagy 202
**miR-31**	Promotes tumor cell migration and invasion 121	Promotes periinfarct angiogenesis and cardiac function recovery 170	Promotes neuronal survival and functional neurological recovery 204
**miR-98**	Promotes chemotherapy resistance 132	Promotes cardiomyocyte survival; induces cardiac function recovery; induces antiinflammation (reduced macrophage infiltration) 143	Promotes neuronal survival and antiinflammation (reduced microglial phagocytosis) 192
**miR-125**	Promotes tumor cell proliferation; induces immune tolerance via M2 macrophage polarization 95	Promotes cardiomyocyte survival and cardiac function recovery 159	Not assessed
**miR-126**	Promotes tumor cell proliferation, angiogenesis and growth in some tumors 129, 131; opposite effects in other tumors 130	Promotes cardiomyocyte survival and cardiac function recovery; induces antiinflammation (proinflammatory cytokines reduced) and antifibrosis; promotes periinfarct angiogenesis 160, 176	Promotes neuronal survival and functional neurological recovery; induces anti-inflammation (reduced microglial activation); induces periinfarct neurogenesis and angiogenesis; induces neuronal (=axonal) plasticity and myelin remodeling 187, 190, 198
**miR-133**	[miR-133a agomir reduces tumor cell proliferation, survival, migration and epithelial-mesenchymal transition; miR-133a antagomir promotes tumor cell survival and migration 238; EV-associated circ-133, a miR-133a sponge, promotes tumor cell migration and metastasis 136	Promotes cardiomyocyte survival and cardiac function recovery 162	Promotes neuronal (=axonal) plasticity and functional neurological recovery 195, 196
**miR-135**	Promotes tumor-associated angiogenesis 101	[miR-135a overexpression promotes cardiomyocyte survival and cardiac function recovery and induces antiinflammation via TLR4 downregulation 272	Promotes neuronal survival; induces antiinflammation (reduced proinflammatory cytokines); inhibits autophagy 210
**miR-146**	Promotes tumor cell proliferation and tumor growth 128	Promotes cardiomyocyte survival; induces antiinflammation (reduced leukocyte infiltration) and antifibrosis 163	Promotes neuronal survival; induces antiinflammation (reduced microglial activation); promotes functional neurological recovery 213
**miR-181**	Promotes tumor cell proliferation; induces immune tolerance via M2 macrophage polarization 95	Not assessed	Promotes astrocyte survival; inhibits astrocytic inflammatory response; promotes periinfarct angiogenesis 214, 220
**miR-210**	Promotes tumor cell migration and invasion; increases tumor-associated angiogenesis 97, 107	Promotes cardiomyocyte survival, cardiac function recovery and angiogenesis 177-179	Promotes angiogenesis and animal survival 221
**miR-223**	Induces chemotherapy resistance 113	Promotes angiogenesis; induces antiinflammation and antifibrosis 180	Promotes neuronal survival; induces antiinflammation (M2 microglia polarization); promotes functional neurological recovery 216
**miR-328**	Promotes tumor cell proliferation, migration, invasion and epithelial - mesenchymal transition; promotes tumor growth 105	Augments cardiomyocyte death and apoptosis 148	[miR-328-3p agomir augments neuronal death, neurological deficits, brain neutrophil invasion and proinflammatory cytokine levels 239
**miR-361**	Promotes tumor cell proliferation and survival; promotes tumor growth 103	[Cardiac-specific miR-361 overexpression reduces cardiomyocyte survival and increases mitochondrial fission; miR361 has knockdown with opposite effects 240	Promotes neuronal survival 217
**miR-494**	Promotes tumor-associated angiogenesis and tumor growth 109	Promotes periinfarct angiogenesis 173	[miR-494 agomir promotes neuronal survival, axonal plasticity and neurological recovery via HDAC3 downregulation 193; miR-494 antagomir promotes neuronal survival and neurological recovery by reducing Th1 helper cell shift and decreasing brain neutrophil infiltrates via HDAC2 upregulation 194, 243

For ncRNAs without studies examining the role of EV-associated ncRNAs, data from ncRNA agomir, antagomir, overexpression or knockdown studies are shown in brackets in the table. **Abbreviations:** EVs, extracellular vesicles; lncR, long non-coding RNA; miR, microRNA.

**Table 5:** EV-associated non-coding RNAs as biomarkers in the three hypoxic conditions.

Author, year, reference	Disease	EV provenance	Non-coding RNA	Abundance	Clinical significance
Bjornetro et al., 2019 253	Locally advanced rectal carcinoma	Plasma	miR-486-5p	Downregulated	Associated with tumor invasion and lymph node metastasis
Bjornetro et al., 2019 253	Locally advanced rectal carcinoma	Plasma	miR-181a-5p	Downregulated	Associated with tumor invasion and lymph node metastasis
Bjornetro et al., 2019 253	Locally advanced rectal carcinoma	Plasma	miR-30d-5p	Upregulated	Associated with tumor metastasis
Zhang et al., 2019 254	Non-small cell lung carcinoma	Serum	lncR MALAT-1	Upregulated	Associated with cell proliferation and migration
Rong et al., 2020 255	Non-small cell lung carcinoma	Serum	lncR MALAT-1	Upregulated	Associated with cancer pathology
Wang et al., 2018 256	Pancreatic carcinoma	Serum	miR-301a	Upregulated	Associated with cancer metastasis
Qeu et al. 2013 248	Pancreatic carcinoma	Serum	miR-17-5p	Upregulated	Associated with cancer pathology
Qeu et al. 2013 248	Pancreatic carcinoma	Serum	miR-21	Upregulated	Associated with cancer pathology
Li et al. 2018 250	Pancreatic ductal adenocarcinoma	Plasma	circPDE8A	Upregulated	Associated with lymphatic invasion, TNM stage and poor survival rate
Zhu et al., 2019 113	Ovarian carcinoma	Serum	miR-223	Upregulated	Associated with cancer recurrence
Ye et al., 2016 110	Nasopharyngeal carcinoma	Plasma	miR-24-3p	Upregulated	Associated with disease-free survival
Hsu et al., 2017 108	Lung carcinoma	Serum	miR-23a	Upregulated	Associated with cancer pathology
Li et al., 2016 114	Oral squamous cell carcinoma	Serum	miR-21	Upregulated	Associated with T stage and N stage
Xue et al., 2017 102	Bladder carcinoma	Serum	lncR UCA1	Upregulated	Associated with cancer pathology
Zhou et al., 2021 257	Breast carcinoma	Serum	lncR NEAT1	Upregulated	Associated with cancer pathology
Zheng et al., 2020 258	Myocardial infarction	Plasma	lncR ENST00000556899.1	Upregulated	Associated with myocardial infarction pathology
Zheng et al., 2020 258	Myocardial infarction	Plasma	lncR ENST00000575985.1	Upregulated	Associated with myocardial infarction pathology, inflammatory markers, disease severity and prognosis
Sun et al., 2020 259	Myocardial infarction	Plasma	lncR UCA1	Upregulated	Associated with myocardial infarction pathology
Chen et al., 2020 260	Myocardial infarction	Serum	lncR NEAT1	Upregulated	Associated with myocardial infarction pathology
Chen et al., 2020 260	Myocardial infarction	Serum	miR-204	Downregulated	Associated with myocardial infarction pathology
Ling et al., 2020 261	Myocardial infarction	Serum	miR-122-5p	Upregulated	Associated with myocardial infarction pathology
Ling et al., 2020 262	Myocardial infarction	Serum	miR-126	Upregulated	Associated with myocardial infarction pathology and disease severity
Ling et al., 2020 262	Myocardial infarction	Serum	miR-21	Upregulated	Associated with myocardial infarction pathology
Su et al., 2019 249	Myocardial infarction	Serum	miR-1915-3p, miR-4507 and miR-3656	Downregulated	Associated with myocardial infarction pathology
Zhou et al., 2018 252	Ischemic stroke	Serum	miR‐134	Upregulated	Associated with ischemic stroke pathology, severity and prognosis
Wang et al., 2018 263	Ischemic stroke	Plasma	miR‐21‐5p	Upregulated	Associated with ischemic stroke pathology
Wang et al., 2018 263	Ischemic stroke	Plasma	miR‐30a‐5p	Upregulated	Associated with ischemic stroke pathology
Chen et al., 2017 264	Ischemic stroke	Serum	miR‐223	Upregulated	Associated with ischemic stroke pathology, severity, and short-term outcome
Li et al., 2017 265	Ischemic stroke	Plasma	miR‐422a	Downregulated	Associated with ischemic stroke pathology
Li et al., 2017 265	Ischemic stroke	Plasma	miR‐125b‐2‐3p	Downregulated	Associated with ischemic stroke pathology
Ji et al., 2016 266	Ischemic stroke	Serum	miR‐9	Upregulated	Associated with ischemic stroke pathology and severity
Ji et al., 2016 266	Ischemic stroke	Serum	miR‐124	Upregulated	Associated with ischemic stroke pathology and severity

## References

[1] Eltzschig HK, Carmeliet P (2011). Hypoxia and inflammation. N Engl J Med.

[2] Eltzschig HK, Eckle T (2011). Ischemia and reperfusion-from mechanism to translation. Nat Med.

[3] Benjamin EJ, Muntner P, Alonso A, Bittencourt MS, Callaway CW, Carson AP (2019). Heart Disease and Stroke Statistics-2019 Update: A Report From the American Heart Association. Circulation.

[4] Wenger RH (2000). Mammalian oxygen sensing, signalling and gene regulation. J Exp Biol.

[5] Fandrey J, Gassmann M (2009). Oxygen sensing and the activation of the hypoxia inducible factor 1 (HIF-1)-invited article. Adv Exp Med Biol.

[6] Wenger RH (2002). Cellular adaptation to hypoxia: O2-sensing protein hydroxylases, hypoxia-inducible transcription factors, and O2-regulated gene expression. Faseb j.

[7] de Heer EC, Jalving M, Harris AL (2020). HIFs, angiogenesis, and metabolism: elusive enemies in breast cancer. J Clin Invest.

[8] Gilkes DM, Semenza GL, Wirtz D (2014). Hypoxia and the extracellular matrix: drivers of tumour metastasis. Nat Rev Cancer.

[9] Li S, Hafeez A, Noorulla F, Geng X, Shao G, Ren C (2017). Preconditioning in neuroprotection: From hypoxia to ischemia. Prog Neurobiol.

[10] Knutson AK, Williams AL, Boisvert WA, Shohet RV (2021). HIF in the heart: development, metabolism, ischemia, and atherosclerosis. J Clin Invest.

[11] Bister N, Pistono C, Huremagic B, Jolkkonen J, Giugno R, Malm T (2020). Hypoxia and extracellular vesicles: A review on methods, vesicular cargo and functions. J Extracell Vesicles.

[12] Choudhry H, Harris AL (2018). Advances in Hypoxia-Inducible Factor Biology. Cell Metab.

[13] van Niel G, D'Angelo G, Raposo G (2018). Shedding light on the cell biology of extracellular vesicles. Nat Rev Mol Cell Biol.

[14] Anastasiadou E, Jacob L, Slack F (2018). Non-coding RNA networks in cancer. Nature reviews Cancer.

[15] Hermann DM, Doeppner TR, Giebel B (2021). Circulating MicroRNAs: Posttranscriptional Regulators and Disease Markers Holding Promise in Stroke Prediction. Stroke.

[16] Wei J, Huang K, Yang C, Kang C (2017). Non-coding RNAs as regulators in epigenetics (Review). Oncology reports.

[17] Lai RC, Tan SS, Yeo RW, Choo AB, Reiner AT, Su Y (2016). MSC secretes at least 3 EV types each with a unique permutation of membrane lipid, protein and RNA. J Extracell Vesicles.

[18] Li C, Xu X (2019). Biological functions and clinical applications of exosomal non-coding RNAs in hepatocellular carcinoma. Cellular and molecular life sciences: CMLS.

[19] Zhang C, Yang X, Qi Q, Gao Y, Wei Q, Han S (2018). lncRNA-HEIH in serum and exosomes as a potential biomarker in the HCV-related hepatocellular carcinoma. Cancer biomarkers: section A of Disease markers.

[20] Kalani M, Alsop E, Meechoovet B, Beecroft T, Agrawal K, Whitsett T (2020). Extracellular microRNAs in blood differentiate between ischaemic and haemorrhagic stroke subtypes. Journal of extracellular vesicles.

[21] Ghoreishy A, Khosravi A, Ghaemmaghami A (2019). Exosomal microRNA and stroke: A review. Journal of cellular biochemistry.

[22] Soekmadji C, Li B, Huang Y, Wang H, An T, Liu C (2020). The future of Extracellular Vesicles as Theranostics - an ISEV meeting report. Journal of extracellular vesicles.

[23] Urabe F, Patil K, Ramm G, Ochiya T, Soekmadji C (2021). Extracellular vesicles in the development of organ-specific metastasis. Journal of extracellular vesicles.

[24] Simpson RJ, Kalra H, Mathivanan S (2012). ExoCarta as a resource for exosomal research. Journal of extracellular vesicles.

[25] Thery C, Ostrowski M, Segura E (2009). Membrane vesicles as conveyors of immune responses. Nature reviews Immunology.

[26] van Niel G, D'Angelo G, Raposo G (2018). Shedding light on the cell biology of extracellular vesicles. Nature reviews Molecular cell biology.

[27] Crescitelli R, Lässer C, Szabó T, Kittel A, Eldh M, Dianzani I (2013). Distinct RNA profiles in subpopulations of extracellular vesicles: apoptotic bodies, microvesicles and exosomes. Journal of extracellular vesicles.

[28] Le Saux S, Aubert-Pouëssel A, Mohammed K, Martineau P, Guglielmi L, Devoisselle J Interest of extracellular vesicles in regards to lipid nanoparticle based systems for intracellular protein delivery. Advanced drug delivery reviews. 2021: 113837.

[29] Battistelli M, Falcieri E (2020). Apoptotic Bodies: Particular Extracellular Vesicles Involved in Intercellular Communication. Biology.

[30] Doyle L, Wang M (2019). Overview of Extracellular Vesicles, Their Origin, Composition, Purpose, and Methods for Exosome Isolation and Analysis. Cells.

[31] Gould SJ, Raposo G (2013). As we wait: coping with an imperfect nomenclature for extracellular vesicles. J Extracell Vesicles.

[32] Thery C, Witwer KW, Aikawa E, Alcaraz MJ, Anderson JD, Andriantsitohaina R (2018). Minimal information for studies of extracellular vesicles 2018 (MISEV2018): a position statement of the International Society for Extracellular Vesicles and update of the MISEV2014 guidelines. J Extracell Vesicles.

[33] Karimi N, Cvjetkovic A, Jang SC, Crescitelli R, Hosseinpour Feizi MA, Nieuwland R (2018). Detailed analysis of the plasma extracellular vesicle proteome after separation from lipoproteins. Cell Mol Life Sci.

[34] Droste M, Tertel T, Jeruschke S, Dittrich R, Kontopoulou E, Walkenfort B (2021). Single Extracellular Vesicle Analysis Performed by Imaging Flow Cytometry and Nanoparticle Tracking Analysis Evaluate the Accuracy of Urinary Extracellular Vesicle Preparation Techniques Differently. Int J Mol Sci.

[35] Sokolova V, Ludwig AK, Hornung S, Rotan O, Horn PA, Epple M (2011). Characterisation of exosomes derived from human cells by nanoparticle tracking analysis and scanning electron microscopy. Colloids Surf B Biointerfaces.

[36] Dragovic RA, Gardiner C, Brooks AS, Tannetta DS, Ferguson DJ, Hole P (2011). Sizing and phenotyping of cellular vesicles using Nanoparticle Tracking Analysis. Nanomedicine.

[37] Fabbiano F, Corsi J, Gurrieri E, Trevisan C, Notarangelo M, D'Agostino VG (2020). RNA packaging into extracellular vesicles: An orchestra of RNA-binding proteins?. J Extracell Vesicles.

[38] Mori MA, Ludwig RG, Garcia-Martin R, Brandão BB, Kahn CR (2019). Extracellular miRNAs: From Biomarkers to Mediators of Physiology and Disease. Cell Metab.

[39] Kapranov P, Cheng J, Dike S, Nix DA, Duttagupta R, Willingham AT (2007). RNA maps reveal new RNA classes and a possible function for pervasive transcription. Science.

[40] Mohapatra S, Pioppini C, Ozpolat B, Calin G (2021). Non-coding RNAs regulation of macrophage polarization in cancer. Molecular cancer.

[41] Lund E, Dahlberg JE (2006). Substrate selectivity of exportin 5 and Dicer in the biogenesis of microRNAs. Cold Spring Harb Symp Quant Biol.

[42] Rana TM (2007). Illuminating the silence: understanding the structure and function of small RNAs. Nat Rev Mol Cell Biol.

[43] Guo H, Ingolia N, Weissman J, Bartel D (2010). Mammalian microRNAs predominantly act to decrease target mRNA levels. Nature.

[44] Bartel DP (2009). MicroRNAs: target recognition and regulatory functions. Cell.

[45] Fabian MR, Sonenberg N, Filipowicz W (2010). Regulation of mRNA translation and stability by microRNAs. Annu Rev Biochem.

[46] Fromm B, Billipp T, Peck LE, Johansen M, Tarver JE, King BL (2015). A Uniform System for the Annotation of Vertebrate microRNA Genes and the Evolution of the Human microRNAome. Annu Rev Genet.

[47] Friedman RC, Farh KK, Burge CB, Bartel DP (2009). Most mammalian mRNAs are conserved targets of microRNAs. Genome Res.

[48] Mack GS (2007). MicroRNA gets down to business. Nat Biotechnol.

[49] Elbashir SM, Harborth J, Lendeckel W, Yalcin A, Weber K, Tuschl T (2001). Duplexes of 21-nucleotide RNAs mediate RNA interference in cultured mammalian cells. Nature.

[50] Carthew RW, Sontheimer EJ (2009). Origins and Mechanisms of miRNAs and siRNAs. Cell.

[51] Siomi MC, Sato K, Pezic D, Aravin AA (2011). PIWI-interacting small RNAs: the vanguard of genome defence. Nat Rev Mol Cell Biol.

[52] Ransohoff JD, Wei Y, Khavari PA (2018). The functions and unique features of long intergenic non-coding RNA. Nat Rev Mol Cell Biol.

[53] Fernandes JCR, Acuña SM, Aoki JI, Floeter-Winter LM, Muxel SM (2019). Long Non-Coding RNAs in the Regulation of Gene Expression: Physiology and Disease. Noncoding RNA.

[54] Wu P, Mo Y, Peng M, Tang T, Zhong Y, Deng X (2020). Emerging role of tumor-related functional peptides encoded by lncRNA and circRNA. Molecular cancer.

[55] Ashwal-Fluss R, Meyer M, Pamudurti N, Ivanov A, Bartok O, Hanan M (2014). circRNA biogenesis competes with pre-mRNA splicing. Molecular cell.

[56] Meng S, Zhou H, Feng Z, Xu Z, Tang Y, Li P (2017). CircRNA: functions and properties of a novel potential biomarker for cancer. Molecular cancer.

[57] O'Brien K, Breyne K, Ughetto S, Laurent L, Breakefield X (2020). RNA delivery by extracellular vesicles in mammalian cells and its applications. Nature reviews Molecular cell biology.

[58] Kogure A, Kosaka N, Ochiya T (2019). Cross-talk between cancer cells and their neighbors via miRNA in extracellular vesicles: an emerging player in cancer metastasis. Journal of biomedical science.

[59] Zheng D, Huo M, Li B, Wang W, Piao H, Wang Y (2020). The Role of Exosomes and Exosomal MicroRNA in Cardiovascular Disease. Frontiers in cell and developmental biology.

[60] Mateescu B, Kowal E, van Balkom B, Bartel S, Bhattacharyya S, Buzás E (2017). Obstacles and opportunities in the functional analysis of extracellular vesicle RNA - an ISEV position paper. Journal of extracellular vesicles.

[61] Khvorova A, Kwak Y, Tamkun M, Majerfeld I, Yarus M (1999). RNAs that bind and change the permeability of phospholipid membranes. Proceedings of the National Academy of Sciences of the United States of America.

[62] Janas T, Janas M, Sapoń K, Janas T (2015). Mechanisms of RNA loading into exosomes. FEBS letters.

[63] Koppers-Lalic D, Hackenberg M, Bijnsdorp I, van Eijndhoven M, Sadek P, Sie D (2014). Nontemplated nucleotide additions distinguish the small RNA composition in cells from exosomes. Cell reports.

[64] Lee Y, Pressman S, Andress A, Kim K, White J, Cassidy J (2009). Silencing by small RNAs is linked to endosomal trafficking. Nature cell biology.

[65] Trajkovic K, Hsu C, Chiantia S, Rajendran L, Wenzel D, Wieland F (2008). Ceramide triggers budding of exosome vesicles into multivesicular endosomes. Science.

[66] Hagemann N, Mohamud Yusuf A, Martiny C, Zhang X, Kleinschnitz C, Gunzer M (2020). Homozygous Smpd1 deficiency aggravates brain ischemia/ reperfusion injury by mechanisms involving polymorphonuclear neutrophils, whereas heterozygous Smpd1 deficiency protects against mild focal cerebral ischemia. Basic Res Cardiol.

[67] Gulbins E, Kolesnick R (2003). Raft ceramide in molecular medicine. Oncogene.

[68] Mohamud Yusuf A, Hagemann N, Hermann DM (2019). The Acid Sphingomyelinase/ Ceramide System as Target for Ischemic Stroke Therapies. Neurosignals.

[69] Janas T, Janas T, Yarus M (2006). Specific RNA binding to ordered phospholipid bilayers. Nucleic Acids Res.

[70] Janas T, Janas T (2011). The selection of aptamers specific for membrane molecular targets. Cell Mol Biol Lett.

[71] Di Liegro C, Schiera G, Di Liegro I (2014). Regulation of mRNA transport, localization and translation in the nervous system of mammals (Review). International journal of molecular medicine.

[72] Gerstberger S, Hafner M, Ascano M, Tuschl T (2014). Evolutionary conservation and expression of human RNA-binding proteins and their role in human genetic disease. Advances in experimental medicine and biology.

[73] Villarroya-Beltri C, Gutiérrez-Vázquez C, Sánchez-Cabo F, Pérez-Hernández D, Vázquez J, Martin-Cofreces N (2013). Sumoylated hnRNPA2B1 controls the sorting of miRNAs into exosomes through binding to specific motifs. Nature communications.

[74] Santangelo L, Giurato G, Cicchini C, Montaldo C, Mancone C, Tarallo R (2016). The RNA-Binding Protein SYNCRIP Is a Component of the Hepatocyte Exosomal Machinery Controlling MicroRNA Sorting. Cell reports.

[75] Shurtleff M, Temoche-Diaz M, Karfilis K, Ri S, Schekman R (2016). Y-box protein 1 is required to sort microRNAs into exosomes in cells and in a cell-free reaction. eLife.

[76] Frank F, Sonenberg N, Nagar B (2010). Structural basis for 5'-nucleotide base-specific recognition of guide RNA by human AGO2. Nature.

[77] Guduric-Fuchs J, O'Connor A, Camp B, O'Neill C, Medina R, Simpson D (2012). Selective extracellular vesicle-mediated export of an overlapping set of microRNAs from multiple cell types. BMC genomics.

[78] Tian T, Zhu Y, Zhou Y, Liang G, Wang Y, Hu F (2014). Exosome uptake through clathrin-mediated endocytosis and macropinocytosis and mediating miR-21 delivery. The Journal of biological chemistry.

[79] Fan Q, Yang L, Zhang X, Peng X, Wei S, Su D (2018). The emerging role of exosome-derived non-coding RNAs in cancer biology. Cancer letters.

[80] Christianson H, Svensson K, van Kuppevelt T, Li J, Belting M (2013). Cancer cell exosomes depend on cell-surface heparan sulfate proteoglycans for their internalization and functional activity. Proceedings of the National Academy of Sciences of the United States of America.

[81] Hurwitz SN, Meckes DG Jr (2019). Extracellular Vesicle Integrins Distinguish Unique Cancers. Proteomes.

[82] Arraud N, Linares R, Tan S, Gounou C, Pasquet JM, Mornet S (2014). Extracellular vesicles from blood plasma: determination of their morphology, size, phenotype and concentration. Journal of Thrombosis and Haemostasis.

[83] Miyanishi M, Tada K, Koike M, Uchiyama Y, Kitamura T, Nagata S (2007). Identification of Tim4 as a phosphatidylserine receptor. Nature.

[84] Hung ME, Leonard JN (2016). A platform for actively loading cargo RNA to elucidate limiting steps in EV-mediated delivery. J Extracell Vesicles.

[85] Németh K, Varga Z, Lenzinger D, Visnovitz T, Koncz A, Hegedűs N (2021). Extracellular vesicle release and uptake by the liver under normo- and hyperlipidemia. Cell Mol Life Sci.

[86] Thery C, Amigorena S, Raposo G, Clayton A (2006). Isolation and characterization of exosomes from cell culture supernatants and biological fluids. Curr Protoc Cell Biol.

[87] Arroyo JD, Chevillet JR, Kroh EM, Ruf IK, Pritchard CC, Gibson DF (2011). Argonaute2 complexes carry a population of circulating microRNAs independent of vesicles in human plasma. Proceedings of the National Academy of Sciences of the United States of America.

[88] Simonsen JB (2017). What Are We Looking At? Extracellular Vesicles, Lipoproteins, or Both?. Circ Res.

[89] Kumar A, Deep G (2020). Exosomes in hypoxia-induced remodeling of the tumor microenvironment. Cancer letters.

[90] Eales K, Hollinshead K, Tennant D (2016). Hypoxia and metabolic adaptation of cancer cells. Oncogenesis.

[91] Yaghoubi S, Najminejad H, Dabaghian M, Karimi M, Abdollahpour-Alitappeh M, Rad F (2020). How hypoxia regulate exosomes in ischemic diseases and cancer microenvironment?. IUBMB life.

[92] Pathania A, Challagundla K (2021). Exosomal Long Non-coding RNAs: Emerging Players in the Tumor Microenvironment. Molecular therapy Nucleic acids.

[93] Guo X, Qiu W, Liu Q, Qian M, Wang S, Zhang Z (2018). Immunosuppressive effects of hypoxia-induced glioma exosomes through myeloid-derived suppressor cells via the miR-10a/Rora and miR-21/Pten Pathways. Oncogene.

[94] Dong C, Liu X, Wang H, Li J, Dai L, Li J (2019). Hypoxic non-small-cell lung cancer cell-derived exosomal miR-21 promotes resistance of normoxic cell to cisplatin. OncoTargets and therapy.

[95] Chen X, Zhou J, Li X, Wang X, Lin Y, Wang X (2018). Exosomes derived from hypoxic epithelial ovarian cancer cells deliver microRNAs to macrophages and elicit a tumor-promoted phenotype. Cancer letters.

[96] Tang T, Yang Z, Zhu Q, Wu Y, Sun K, Alahdal M Up-regulation of miR-210 induced by a hypoxic microenvironment promotes breast cancer stem cells metastasis, proliferation, and self-renewal by targeting E-cadherin. FASEB journal: official publication of the Federation of American Societies for Experimental Biology. 2018: fj201801013R.

[97] Zhang X, Sai B, Wang F, Wang L, Wang Y, Zheng L (2019). Hypoxic BMSC-derived exosomal miRNAs promote metastasis of lung cancer cells via STAT3-induced EMT. Molecular cancer.

[98] Yu Y, Min Z, Zhou Zhihang, Linhong M, Tao R, Yan L (2019). Hypoxia-induced exosomes promote hepatocellular carcinoma proliferation and metastasis via miR-1273f transfer. Experimental cell research.

[99] Takahashi K, Yan I, Kogure T, Haga H, Patel T (2014). Extracellular vesicle-mediated transfer of long non-coding RNA ROR modulates chemosensitivity in human hepatocellular cancer. FEBS open bio.

[100] Park J, Dutta B, Tse S, Gupta N, Tan C, Low J (2019). Hypoxia-induced tumor exosomes promote M2-like macrophage polarization of infiltrating myeloid cells and microRNA-mediated metabolic shift. Oncogene.

[101] Umezu T, Tadokoro H, Azuma K, Yoshizawa S, Ohyashiki K, Ohyashiki J (2014). Exosomal miR-135b shed from hypoxic multiple myeloma cells enhances angiogenesis by targeting factor-inhibiting HIF-1. Blood.

[102] Xue M, Chen W, Xiang A, Wang R, Chen H, Pan J (2017). Hypoxic exosomes facilitate bladder tumor growth and development through transferring long non-coding RNA-UCA1. Molecular cancer.

[103] Li J, Yang P, Chen F, Tan Y, Huang C, Shen H (2021). Hypoxic colorectal cancer-derived extracellular vesicles deliver microRNA-361-3p to facilitate cell proliferation by targeting TRAF3 via the noncanonical NF-κB pathways. Clinical and translational medicine.

[104] Meng L, Xing Z, Guo Z, Qiu Y, Liu Z (2021). Hypoxia-induced microRNA-155 overexpression in extracellular vesicles promotes renal cell carcinoma progression by targeting FOXO3. Aging (Albany NY).

[105] Liu X, Jiang F, Wang Z, Tang L, Zou B, Xu P (2021). Hypoxic bone marrow mesenchymal cell-extracellular vesicles containing miR-328-3p promote lung cancer progression via the NF2-mediated Hippo axis. J Cell Mol Med.

[106] Guo Z, Wang X, Yang Y, Chen W, Zhang K, Teng B (2020). Hypoxic Tumor-Derived Exosomal Long Noncoding RNA UCA1 Promotes Angiogenesis via miR-96-5p/AMOTL2 in Pancreatic Cancer. Molecular therapy Nucleic acids.

[107] Tadokoro H, Umezu T, Ohyashiki K, Hirano T, Ohyashiki J (2013). Exosomes derived from hypoxic leukemia cells enhance tube formation in endothelial cells. The Journal of biological chemistry.

[108] Hsu Y, Hung J, Chang W, Lin Y, Pan Y, Tsai P (2017). Hypoxic lung cancer-secreted exosomal miR-23a increased angiogenesis and vascular permeability by targeting prolyl hydroxylase and tight junction protein ZO-1. Oncogene.

[109] Mao G, Liu Y, Fang X, Liu Y, Fang L, Lin L (2015). Tumor-derived microRNA-494 promotes angiogenesis in non-small cell lung cancer. Angiogenesis.

[110] Ye S, Zhang H, Cai T, Liu Y, Ni J, He J (2016). Exosomal miR-24-3p impedes T-cell function by targeting FGF11 and serves as a potential prognostic biomarker for nasopharyngeal carcinoma. The Journal of pathology.

[111] Berchem G, Noman M, Bosseler M, Paggetti J, Baconnais S, Le Cam E (2016). Hypoxic tumor-derived microvesicles negatively regulate NK cell function by a mechanism involving TGF-β and miR23a transfer. Oncoimmunology.

[112] Zeng Z, Zhao Y, Chen Q, Zhu S, Niu Y, Ye Z (2021). Hypoxic exosomal HIF-1α-stabilizing circZNF91 promotes chemoresistance of normoxic pancreatic cancer cells via enhancing glycolysis. Oncogene.

[113] Zhu X, Shen H, Yin X, Yang M, Wei H, Chen Q (2019). Macrophages derived exosomes deliver miR-223 to epithelial ovarian cancer cells to elicit a chemoresistant phenotype. Journal of experimental & clinical cancer research: CR.

[114] Li L, Li C, Wang S, Wang Z, Jiang J, Wang W (2016). Exosomes Derived from Hypoxic Oral Squamous Cell Carcinoma Cells Deliver miR-21 to Normoxic Cells to Elicit a Prometastatic Phenotype. Cancer research.

[115] Chen F, Xu B, Li J, Yang X, Gu J, Yao X (2021). Hypoxic tumour cell-derived exosomal miR-340-5p promotes radioresistance of oesophageal squamous cell carcinoma via KLF10. J Exp Clin Cancer Res.

[116] Hsu YL, Hung JY, Chang WA, Jian SF, Lin YS, Pan YC (2018). Hypoxic Lung-Cancer-Derived Extracellular Vesicle MicroRNA-103a Increases the Oncogenic Effects of Macrophages by Targeting PTEN. Mol Ther.

[117] Wang X, Luo G, Zhang K, Cao J, Huang C, Jiang T (2018). Hypoxic Tumor-Derived Exosomal miR-301a Mediates M2 Macrophage Polarization via PTEN/PI3Kγ to Promote Pancreatic Cancer Metastasis. Cancer Res.

[118] Chen X, Ying X, Wang X, Wu X, Zhu Q, Wang X (2017). Exosomes derived from hypoxic epithelial ovarian cancer deliver microRNA-940 to induce macrophage M2 polarization. Oncol Rep.

[119] Li L, Cao B, Liang X, Lu S, Luo H, Wang Z (2019). Microenvironmental oxygen pressure orchestrates an anti- and pro-tumoral γδ T cell equilibrium via tumor-derived exosomes. Oncogene.

[120] Takahashi K, Yan IK, Haga H, Patel T (2014). Modulation of hypoxia-signaling pathways by extracellular linc-RoR. J Cell Sci.

[121] Yu F, Liang M, Huang Y, Wu W, Zheng B, Chen C (2021). Hypoxic tumor-derived exosomal miR-31-5p promotes lung adenocarcinoma metastasis by negatively regulating SATB2-reversed EMT and activating MEK/ERK signaling. J Exp Clin Cancer Res.

[122] Zhou D, Gu J, Wang Y, Wu H, Cheng W, Wang Q (2021). Long non-coding RNA NEAT1 transported by extracellular vesicles contributes to breast cancer development by sponging microRNA-141-3p and regulating KLF12. Cell Biosci.

[123] Zhang R, Xia Y, Wang Z, Zheng J, Chen Y, Li X (2017). Serum long non coding RNA MALAT-1 protected by exosomes is up-regulated and promotes cell proliferation and migration in non-small cell lung cancer. Biochem Biophys Res Commun.

[124] Rong F, Liu L, Zou C, Zeng J, Xu Y (2020). MALAT1 Promotes Cell Tumorigenicity Through Regulating miR-515-5p/EEF2 Axis in Non-Small Cell Lung Cancer. Cancer Manag Res.

[125] Wang S, Wang T, Liu D, Kong H (2020). LncRNA MALAT1 Aggravates the Progression of Non-Small Cell Lung Cancer by Stimulating the Expression of COMMD8 via Targeting miR-613. Cancer Manag Res.

[126] Yang Y, Ma S, Ye Z, Zheng Y, Zheng Z, Liu X (2022). NEAT1 in bone marrow mesenchymal stem cell-derived extracellular vesicles promotes melanoma by inducing M2 macrophage polarization. Cancer Gene Ther.

[127] Yang J, Sun G, Hu Y, Yang J, Shi Y, Liu H (2019). Extracellular Vesicle lncRNA Metastasis-Associated Lung Adenocarcinoma Transcript 1 Released From Glioma Stem Cells Modulates the Inflammatory Response of Microglia After Lipopolysaccharide Stimulation Through Regulating miR-129-5p/High Mobility Group Box-1 Protein Axis. Front Immunol.

[128] Katakowski M, Buller B, Zheng X, Lu Y, Rogers T, Osobamiro O (2013). Exosomes from marrow stromal cells expressing miR-146b inhibit glioma growth. Cancer Lett.

[129] Hu Y, Zai H, Jiang W, Yao Y, Ou Z, Zhu Q (2021). miR-126 in Extracellular Vesicles Derived from Hepatoblastoma Cells Promotes the Tumorigenesis of Hepatoblastoma through Inducing the Differentiation of BMSCs into Cancer Stem Cells. J Immunol Res.

[130] Li M, Wang Q, Zhang X, Yan N, Li X (2020). Exosomal miR-126 blocks the development of non-small cell lung cancer through the inhibition of ITGA6. Cancer Cell Int.

[131] Taverna S, Amodeo V, Saieva L, Russo A, Giallombardo M, De Leo G (2014). Exosomal shuttling of miR-126 in endothelial cells modulates adhesive and migratory abilities of chronic myelogenous leukemia cells. Mol Cancer.

[132] Guo H, Ha C, Dong H, Yang Z, Ma Y, Ding Y (2019). Cancer-associated fibroblast-derived exosomal microRNA-98-5p promotes cisplatin resistance in ovarian cancer by targeting CDKN1A. Cancer Cell Int.

[133] Liu H, Chen W, Zhi X, Chen EJ, Wei T, Zhang J (2018). Tumor-derived exosomes promote tumor self-seeding in hepatocellular carcinoma by transferring miRNA-25-5p to enhance cell motility. Oncogene.

[134] Zeng Z, Li Y, Pan Y, Lan X, Song F, Sun J (2018). Cancer-derived exosomal miR-25-3p promotes pre-metastatic niche formation by inducing vascular permeability and angiogenesis. Nat Commun.

[135] Wang X, Zhang H, Yang H, Bai M, Ning T, Deng T (2020). Exosome-delivered circRNA promotes glycolysis to induce chemoresistance through the miR-122-PKM2 axis in colorectal cancer. Molecular oncology.

[136] Yang H, Zhang H, Yang Y, Wang X, Deng T, Liu R (2020). Hypoxia induced exosomal circRNA promotes metastasis of Colorectal Cancer via targeting GEF-H1/RhoA axis. Theranostics.

[137] Wang Y, Zhang M, Zhou F (2020). Biological functions and clinical applications of exosomal long non-coding RNAs in cancer. Journal of cellular and molecular medicine.

[138] Sun Z, Yang S, Zhou Q, Wang G, Song J, Li Z (2018). Emerging role of exosome-derived long non-coding RNAs in tumor microenvironment. Molecular cancer.

[139] Whiteside T (2016). Exosomes and tumor-mediated immune suppression. The Journal of clinical investigation.

[140] Li K, Bai Y, Li J, Li S, Pan J, Cheng Y (2021). LncRNA HCP5 in hBMSC-derived exosomes alleviates myocardial ischemia reperfusion injury by sponging miR-497 to activate IGF1/PI3K/AKT pathway. International journal of cardiology.

[141] Gu H, Liu Z, Li Y, Xie Y, Yao J, Zhu Y (2018). Serum-Derived Extracellular Vesicles Protect Against Acute Myocardial Infarction by Regulating miR-21/PDCD4 Signaling Pathway. Frontiers in physiology.

[142] Pu L, Kong X, Li H, He X (2021). Exosomes released from mesenchymal stem cells overexpressing microRNA-30e ameliorate heart failure in rats with myocardial infarction. American journal of translational research.

[143] Zhang L, Wei Q, Liu X, Zhang T, Wang S, Zhou L (2021). Exosomal microRNA-98-5p from hypoxic bone marrow mesenchymal stem cells inhibits myocardial ischemia-reperfusion injury by reducing TLR4 and activating the PI3K/Akt signaling pathway. Int Immunopharmacol.

[144] Wu Z, Cheng S, Wang S, Li W, Liu J (2021). BMSCs-derived exosomal microRNA-150-5p attenuates myocardial infarction in mice. Int Immunopharmacol.

[145] Li Y, Zhou J, Zhang O, Wu X, Guan X, Xue Y (2020). Bone marrow mesenchymal stem cells-derived exosomal microRNA-185 represses ventricular remolding of mice with myocardial infarction by inhibiting SOCS2. Int Immunopharmacol.

[146] Wu Y, Peng W, Fang M, Wu M, Wu M (2021). MSCs-Derived Extracellular Vesicles Carrying miR-212-5p Alleviate Myocardial Infarction-Induced Cardiac Fibrosis via NLRC5/VEGF/TGF-β1/SMAD Axis. J Cardiovasc Transl Res.

[147] Zhang C, Shao K, Liu C, Li C, Yu B (2019). Hypoxic preconditioning BMSCs-exosomes inhibit cardiomyocyte apoptosis after acute myocardial infarction by upregulating microRNA-24. European review for medical and pharmacological sciences.

[148] Huang J, Wang F, Sun X, Chu X, Jiang R, Wang Y (2021). Myocardial infarction cardiomyocytes-derived exosomal miR-328-3p promote apoptosis via Caspase signaling. American journal of translational research.

[149] Kenneweg F, Bang C, Xiao K, Boulanger CM, Loyer X, Mazlan S (2019). Long Noncoding RNA-Enriched Vesicles Secreted by Hypoxic Cardiomyocytes Drive Cardiac Fibrosis. Mol Ther Nucleic Acids.

[150] Wang X, Zhu Y, Wu C, Liu W, He Y, Yang Q (2021). Adipose-Derived Mesenchymal Stem Cells-Derived Exosomes Carry MicroRNA-671 to Alleviate Myocardial Infarction Through Inactivating the TGFBR2/Smad2 Axis. Inflammation.

[151] Zhang Z, Buller B, Chopp M (2019). Exosomes - beyond stem cells for restorative therapy in stroke and neurological injury. Nature reviews Neurology.

[152] Zheng X, Hermann D, Bähr M, Doeppner T (2021). The role of small extracellular vesicles in cerebral and myocardial ischemia-Molecular signals, treatment targets, and future clinical translation. Stem cells (Dayton, Ohio).

[153] Diao L, Zhang Q (2021). Transfer of lncRNA UCA1 by hUCMSCs-derived exosomes protects against hypoxia/reoxygenation injury through impairing miR-143-targeted degradation of Bcl-2. Aging (Albany NY).

[154] Shyu K, Wang B, Fang W, Pan C, Lin C (2020). Hyperbaric oxygen-induced long non-coding RNA MALAT1 exosomes suppress MicroRNA-92a expression in a rat model of acute myocardial infarction. Journal of cellular and molecular medicine.

[155] Chen G, Yue A, Wang M, Ruan Z, Zhu L (2021). The Exosomal lncRNA KLF3-AS1 From Ischemic Cardiomyocytes Mediates IGF-1 Secretion by MSCs to Rescue Myocardial Ischemia-Reperfusion Injury. Front Cardiovasc Med.

[156] Mao Q, Liang X, Zhang C, Pang Y, Lu Y (2019). LncRNA KLF3-AS1 in human mesenchymal stem cell-derived exosomes ameliorates pyroptosis of cardiomyocytes and myocardial infarction through miR-138-5p/Sirt1 axis. Stem cell research & therapy.

[157] Song Y, Zhang C, Zhang J, Jiao Z, Dong N, Wang G (2019). Localized injection of miRNA-21-enriched extracellular vesicles effectively restores cardiac function after myocardial infarction. Theranostics.

[158] Zheng S, Wang L, Ma H, Sun F, Wen F (2021). microRNA-129 overexpression in endothelial cell-derived extracellular vesicle influences inflammatory response caused by myocardial ischemia/reperfusion injury. Cell Biol Int.

[159] Zhu LP, Tian T, Wang JY, He JN, Chen T, Pan M (2018). Hypoxia-elicited mesenchymal stem cell-derived exosomes facilitates cardiac repair through miR-125b-mediated prevention of cell death in myocardial infarction. Theranostics.

[160] Luo Q, Guo D, Liu G, Chen G, Hang M, Jin M (2017). Exosomes from MiR-126-Overexpressing Adscs Are Therapeutic in Relieving Acute Myocardial Ischaemic Injury. Cell Physiol Biochem.

[161] Wang S, Dong J, Li L, Wu R, Xu L, Ren Y (2022). Exosomes derived from miR-129-5p modified bone marrow mesenchymal stem cells represses ventricular remolding of mice with myocardial infarction. J Tissue Eng Regen Med.

[162] Zhu W, Sun L, Zhao P, Liu Y, Zhang J, Zhang Y (2021). Macrophage migration inhibitory factor facilitates the therapeutic efficacy of mesenchymal stem cells derived exosomes in acute myocardial infarction through upregulating miR-133a-3p. Journal of nanobiotechnology.

[163] Pan J, Alimujiang M, Chen Q, Shi H, Luo X (2019). Exosomes derived from miR-146a-modified adipose-derived stem cells attenuate acute myocardial infarction-induced myocardial damage via downregulation of early growth response factor 1. J Cell Biochem.

[164] Ke X, Yang R, Wu F, Wang X, Liang J, Hu X (2021). Exosomal miR-218-5p/miR-363-3p from Endothelial Progenitor Cells Ameliorate Myocardial Infarction by Targeting the p53/JMY Signaling Pathway. Oxid Med Cell Longev.

[165] Fu DL, Jiang H, Li CY, Gao T, Liu MR, Li HW (2020). MicroRNA-338 in MSCs-derived exosomes inhibits cardiomyocyte apoptosis in myocardial infarction. Eur Rev Med Pharmacol Sci.

[166] Sánchez-Sánchez R, Gómez-Ferrer M, Reinal I, Buigues M, Villanueva-Bádenas E, Ontoria-Oviedo I (2021). miR-4732-3p in Extracellular Vesicles From Mesenchymal Stromal Cells Is Cardioprotective During Myocardial Ischemia. Frontiers in cell and developmental biology.

[167] Lin B, Chen X, Lu C, Xu J, Qiu Y, Liu X (2021). Loss of exosomal LncRNA HCG15 prevents acute myocardial ischemic injury through the NF-κB/p65 and p38 pathways. Cell Death Dis.

[168] Ning W, Li S, Yang W, Yang B, Xin C, Ping X (2021). Blocking exosomal miRNA-153-3p derived from bone marrow mesenchymal stem cells ameliorates hypoxia-induced myocardial and microvascular damage by targeting the ANGPT1-mediated VEGF/PI3k/Akt/eNOS pathway. Cell Signal.

[169] He Q, Ye A, Ye W, Liao X, Qin G, Xu Y (2021). Cancer-secreted exosomal miR-21-5p induces angiogenesis and vascular permeability by targeting KRIT1. Cell Death Dis.

[170] Zhu D, Wang Y, Thomas M, McLaughlin K, Oguljahan B, Henderson J (2022). Exosomes from adipose-derived stem cells alleviate myocardial infarction via microRNA-31/FIH1/HIF-1α pathway. J Mol Cell Cardiol.

[171] Youn S, Li Y, Kim Y, Sudhahar V, Abdelsaid K, Kim H (2019). Modification of Cardiac Progenitor Cell-Derived Exosomes by miR-322 Provides Protection against Myocardial Infarction through Nox2-Dependent Angiogenesis. Antioxidants (Basel, Switzerland).

[172] Li Q, Xu Y, Lv K, Wang Y, Zhong Z, Xiao C (2021). Small extracellular vesicles containing miR-486-5p promote angiogenesis after myocardial infarction in mice and nonhuman primates. Science translational medicine.

[173] Liu H, Zhang Y, Yuan J, Gao W, Zhong X, Yao K (2021). Dendritic cell-derived exosomal miR-494-3p promotes angiogenesis following myocardial infarction. International journal of molecular medicine.

[174] Climent M, Quintavalle M, Miragoli M, Chen J, Condorelli G, Elia L (2015). TGFβ Triggers miR-143/145 Transfer From Smooth Muscle Cells to Endothelial Cells, Thereby Modulating Vessel Stabilization. Circ Res.

[175] Jiang W, Song Q, Lu Z, Wang S, Liu T, Wang X (2021). Myocardial Infarction-Associated Extracellular Vesicle-Delivered miR-208b Affects the Growth of Human Umbilical Vein Endothelial Cells via Regulating CDKN1A. BioMed research international.

[176] Chen J, Cui C, Yang X, Xu J, Venkat P, Zacharek A (2017). MiR-126 Affects Brain-Heart Interaction after Cerebral Ischemic Stroke. Transl Stroke Res.

[177] Barile L, Lionetti V, Cervio E, Matteucci M, Gherghiceanu M, Popescu LM (2014). Extracellular vesicles from human cardiac progenitor cells inhibit cardiomyocyte apoptosis and improve cardiac function after myocardial infarction. Cardiovasc Res.

[178] Cheng H, Chang S, Xu R, Chen L, Song X, Wu J (2020). Hypoxia-challenged MSC-derived exosomes deliver miR-210 to attenuate post-infarction cardiac apoptosis. Stem Cell Res Ther.

[179] Wang N, Chen C, Yang D, Liao Q, Luo H, Wang X (2017). Mesenchymal stem cells-derived extracellular vesicles, via miR-210, improve infarcted cardiac function by promotion of angiogenesis. Biochim Biophys Acta Mol Basis Dis.

[180] Yang M, Liao M, Liu R, Zhang Q, Zhang S, He Y (2022). Human umbilical cord mesenchymal stem cell-derived extracellular vesicles loaded with miR-223 ameliorate myocardial infarction through P53/S100A9 axis. Genomics.

[181] Peng Y, Zhao JL, Peng ZY, Xu WF, Yu GL (2020). Exosomal miR-25-3p from mesenchymal stem cells alleviates myocardial infarction by targeting pro-apoptotic proteins and EZH2. Cell Death Dis.

[182] Zhu D, Wang Y, Thomas M, McLaughlin K, Oguljahan B, Henderson J (2021). Exosomes from adipose-derived stem cells alleviate myocardial infarction via microRNA-31/FIH1/HIF-1α pathway. Journal of molecular and cellular cardiology.

[183] Hermann DM, Chopp M (2012). Promoting brain remodelling and plasticity for stroke recovery: therapeutic promise and potential pitfalls of clinical translation. Lancet neurology.

[184] Reitmeir R, Kilic E, Kilic U, Bacigaluppi M, ElAli A, Salani G (2011). Post-acute delivery of erythropoietin induces stroke recovery by promoting perilesional tissue remodelling and contralesional pyramidal tract plasticity. Brain: a journal of neurology.

[185] Feng B, Meng L, Luan L, Fang Z, Zhao P, Zhao G (2020). Upregulation of Extracellular Vesicles-Encapsulated miR-132 Released From Mesenchymal Stem Cells Attenuates Ischemic Neuronal Injury by Inhibiting Smad2/c-jun Pathway via Acvr2b Suppression. Front Cell Dev Biol.

[186] Ai Z, Cheng C, Zhou L, Yin S, Wang L, Liu Y (2021). Bone marrow mesenchymal stem cells-derived extracellular vesicles carrying microRNA-221-3p protect against ischemic stroke via ATF3. Brain Res Bull.

[187] Venkat P, Cui C, Chopp M, Zacharek A, Wang F, Landschoot-Ward J (2019). MiR-126 Mediates Brain Endothelial Cell Exosome Treatment-Induced Neurorestorative Effects After Stroke in Type 2 Diabetes Mellitus Mice. Stroke.

[188] Cai G, Cai G, Zhou H, Zhuang Z, Liu K, Pei S (2021). Mesenchymal stem cell-derived exosome miR-542-3p suppresses inflammation and prevents cerebral infarction. Stem Cell Res Ther.

[189] Qi Z, Zhao Y, Su Y, Cao B, Yang J, Xing Q (2021). Serum Extracellular Vesicle-Derived miR-124-3p as a Diagnostic and Predictive Marker for Early-Stage Acute Ischemic Stroke. Frontiers in molecular biosciences.

[190] Geng W, Tang H, Luo S, Lv Y, Liang D, Kang X (2019). Exosomes from miRNA-126-modified ADSCs promotes functional recovery after stroke in rats by improving neurogenesis and suppressing microglia activation. American journal of translational research.

[191] Yang L, Han B, Zhang Z, Wang S, Bai Y, Zhang Y (2020). Extracellular Vesicle-Mediated Delivery of Circular RNA SCMH1 Promotes Functional Recovery in Rodent and Nonhuman Primate Ischemic Stroke Models. Circulation.

[192] Yang J, Cao LL, Wang XP, Guo W, Guo RB, Sun YQ (2021). Neuronal extracellular vesicle derived miR-98 prevents salvageable neurons from microglial phagocytosis in acute ischemic stroke. Cell Death Dis.

[193] Zhao H, Li G, Zhang S, Li F, Wang R, Tao Z (2019). Inhibition of histone deacetylase 3 by MiR-494 alleviates neuronal loss and improves neurological recovery in experimental stroke. J Cereb Blood Flow Metab.

[194] Zhao H, Li G, Wang R, Tao Z, Ma Q, Zhang S (2020). Silencing of microRNA-494 inhibits the neurotoxic Th1 shift via regulating HDAC2-STAT4 cascade in ischaemic stroke. Br J Pharmacol.

[195] Xin H, Li Y, Buller B, Katakowski M, Zhang Y, Wang X (2012). Exosome-mediated transfer of miR-133b from multipotent mesenchymal stromal cells to neural cells contributes to neurite outgrowth. Stem Cells.

[196] Xin H, Li Y, Liu Z, Wang X, Shang X, Cui Y (2013). MiR-133b promotes neural plasticity and functional recovery after treatment of stroke with multipotent mesenchymal stromal cells in rats via transfer of exosome-enriched extracellular particles. Stem Cells.

[197] Li G, Xiao L, Qin H, Zhuang Q, Zhang W, Liu L (2020). Exosomes-carried microRNA-26b-5p regulates microglia M1 polarization after cerebral ischemia/reperfusion. Cell Cycle.

[198] Gregorius J, Wang C, Stambouli O, Hussner T, Qi Y, Tertel T (2021). Small extracellular vesicles obtained from hypoxic mesenchymal stromal cells have unique characteristics that promote cerebral angiogenesis, brain remodeling and neurological recovery after focal cerebral ischemia in mice. Basic Res Cardiol.

[199] Fan B, Chopp M, Zhang ZG, Liu XS (2020). Emerging Roles of microRNAs as Biomarkers and Therapeutic Targets for Diabetic Neuropathy. Front Neurol.

[200] Chen W, Wang H, Zhu Z, Feng J, Chen L (2020). Exosome-Shuttled circSHOC2 from IPASs Regulates Neuronal Autophagy and Ameliorates Ischemic Brain Injury via the miR-7670-3p/SIRT1 Axis. Molecular therapy Nucleic acids.

[201] Zhang Y, Liu J, Su M, Wang X, Xie C (2021). Exosomal microRNA-22-3p alleviates cerebral ischemic injury by modulating KDM6B/BMP2/BMF axis. Stem Cell Res Ther.

[202] Kuang Y, Zheng X, Zhang L, Ai X, Venkataramani V, Kilic E (2020). Adipose-derived mesenchymal stem cells reduce autophagy in stroke mice by extracellular vesicle transfer of miR-25. Journal of extracellular vesicles.

[203] Hou Z, Chen J, Yang H, Hu X, Yang F (2021). microRNA-26a shuttled by extracellular vesicles secreted from adipose-derived mesenchymal stem cells reduce neuronal damage through KLF9-mediated regulation of TRAF2/KLF2 axis. Adipocyte.

[204] Lv H, Li J, Che Y (2021). miR-31 from adipose stem cell-derived extracellular vesicles promotes recovery of neurological function after ischemic stroke by inhibiting TRAF6 and IRF5. Exp Neurol.

[205] Wu W, Liu J, Yang C, Xu Z, Huang J, Lin J (2020). Astrocyte-derived exosome-transported microRNA-34c is neuroprotective against cerebral ischemia/reperfusion injury via TLR7 and the NF-kappaB/MAPK pathways. Brain Res Bull.

[206] Xu L, Cao H, Xie Y, Zhang Y, Du M, Xu X (2019). Exosome-shuttled miR-92b-3p from ischemic preconditioned astrocytes protects neurons against oxygen and glucose deprivation. Brain Res.

[207] Li Z, Song Y, He T, Wen R, Li Y, Chen T (2021). M2 microglial small extracellular vesicles reduce glial scar formation via the miR-124/STAT3 pathway after ischemic stroke in mice. Theranostics.

[208] Cui J, Liu N, Chang Z, Gao Y, Bao M, Xie Y (2020). Exosomal MicroRNA-126 from RIPC Serum Is Involved in Hypoxia Tolerance in SH-SY5Y Cells by Downregulating DNMT3B. Mol Ther Nucleic Acids.

[209] Xiao Y, Geng F, Wang G, Li X, Zhu J, Zhu W (2018). Bone marrow-derived mesenchymal stem cells-derived exosomes prevent oligodendrocyte apoptosis through exosomal miR-134 by targeting caspase-8. J Cell Biochem.

[210] Liu Y, Li YP, Xiao LM, Chen LK, Zheng SY, Zeng EM (2021). Extracellular vesicles derived from M2 microglia reduce ischemic brain injury through microRNA-135a-5p/TXNIP/NLRP3 axis. Lab Invest.

[211] Zhang D, Cai G, Liu K, Zhuang Z, Jia K, Pei S (2021). Microglia exosomal miRNA-137 attenuates ischemic brain injury through targeting Notch1. Aging (Albany NY).

[212] Deng Y, Chen D, Gao F, Lv H, Zhang G, Sun X (2019). Exosomes derived from microRNA-138-5p-overexpressing bone marrow-derived mesenchymal stem cells confer neuroprotection to astrocytes following ischemic stroke via inhibition of LCN2. J Biol Eng.

[213] Zhang Z, Zou X, Zhang R, Xie Y, Feng Z, Li F (2021). Human umbilical cord mesenchymal stem cell-derived exosomal miR-146a-5p reduces microglial-mediated neuroinflammation via suppression of the IRAK1/TRAF6 signaling pathway after ischemic stroke. Aging.

[214] Song H, Zhang X, Chen R, Miao J, Wang L, Cui L (2019). Cortical Neuron-Derived Exosomal MicroRNA-181c-3p Inhibits Neuroinflammation by Downregulating CXCL1 in Astrocytes of a Rat Model with Ischemic Brain Injury. Neuroimmunomodulation.

[215] Zhong Y, Luo L (2021). Exosomes from Human Umbilical Vein Endothelial Cells Ameliorate Ischemic Injuries by Suppressing the RNA Component of Mitochondrial RNA-processing Endoribonuclease via the Induction of miR-206/miR-1-3p Levels. Neuroscience.

[216] Zhao Y, Gan Y, Xu G, Hua K, Liu D (2020). Exosomes from MSCs overexpressing microRNA-223-3p attenuate cerebral ischemia through inhibiting microglial M1 polarization mediated inflammation. Life sciences.

[217] Bu X, Li D, Wang F, Sun Q, Zhang Z (2020). Protective Role of Astrocyte-Derived Exosomal microRNA-361 in Cerebral Ischemic-Reperfusion Injury by Regulating the AMPK/mTOR Signaling Pathway and Targeting CTSB. Neuropsychiatr Dis Treat.

[218] Yue KY, Zhang PR, Zheng MH, Cao XL, Cao Y, Zhang YZ (2019). Neurons can upregulate Cav-1 to increase intake of endothelial cells-derived extracellular vesicles that attenuate apoptosis via miR-1290. Cell Death Dis.

[219] Ye Z, Hu J, Xu H, Sun B, Jin Y, Zhang Y (2021). Serum Exosomal microRNA-27-3p Aggravates Cerebral Injury and Inflammation in Patients with Acute Cerebral Infarction by Targeting PPARγ. Inflammation.

[220] Yang Y, Cai Y, Zhang Y, Liu J, Xu Z (2018). Exosomes Secreted by Adipose-Derived Stem Cells Contribute to Angiogenesis of Brain Microvascular Endothelial Cells Following Oxygen-Glucose Deprivation In Vitro Through MicroRNA-181b/TRPM7 Axis. J Mol Neurosci.

[221] Zhang H, Wu J, Wu J, Fan Q, Zhou J, Wu J (2019). Exosome-mediated targeted delivery of miR-210 for angiogenic therapy after cerebral ischemia in mice. Journal of nanobiotechnology.

[222] Xin H, Katakowski M, Wang F, Qian JY, Liu XS, Ali MM (2017). MicroRNA cluster miR-17-92 Cluster in Exosomes Enhance Neuroplasticity and Functional Recovery After Stroke in Rats. Stroke.

[223] Ling X, Zhang G, Xia Y, Zhu Q, Zhang J, Li Q (2020). Exosomes from human urine-derived stem cells enhanced neurogenesis via miR-26a/HDAC6 axis after ischaemic stroke. J Cell Mol Med.

[224] Yang J, Zhang X, Chen X, Wang L, Yang G (2017). Exosome Mediated Delivery of miR-124 Promotes Neurogenesis after Ischemia. Mol Ther Nucleic Acids.

[225] El Bassit G, Patel RS, Carter G, Shibu V, Patel AA, Song S (2017). MALAT1 in Human Adipose Stem Cells Modulates Survival and Alternative Splicing of PKCδII in HT22 Cells. Endocrinology.

[226] Wang Z, Li X, Huang L, Liu G, Chen Y, Li B (2020). Long Non-coding RNAs (lncRNAs), A New Target in Stroke. Cellular and molecular neurobiology.

[227] Xu Z, Yan Y, Qian L, Gong Z (2017). Long non-coding RNAs act as regulators of cell autophagy in diseases (Review). Oncology reports.

[228] Xing H, Tan J, Miao Y, Lv Y, Zhang Q (2021). Crosstalk between exosomes and autophagy: A review of molecular mechanisms and therapies. Journal of cellular and molecular medicine.

[229] Ajoolabady A, Wang S, Kroemer G, Penninger J, Uversky V, Pratico D (2021). Targeting autophagy in ischemic stroke: From molecular mechanisms to clinical therapeutics. Pharmacology & therapeutics.

[230] Han B, Zhang Y, Zhang Y, Bai Y, Chen X, Huang R (2018). Novel insight into circular RNA HECTD1 in astrocyte activation via autophagy by targeting MIR142-TIPARP: implications for cerebral ischemic stroke. Autophagy.

[231] Bacigaluppi M, Russo GL, Peruzzotti-Jametti L, Rossi S, Sandrone S, Butti E (2016). Neural Stem Cell Transplantation Induces Stroke Recovery by Upregulating Glutamate Transporter GLT-1 in Astrocytes. J Neurosci.

[232] Liu Z, Li Y, Zhang ZG, Cui X, Cui Y, Lu M (2010). Bone marrow stromal cells enhance inter- and intracortical axonal connections after ischemic stroke in adult rats. J Cereb Blood Flow Metab.

[233] Bassett A, Azzam G, Wheatley L, Tibbit C, Rajakumar T, McGowan S (2014). Understanding functional miRNA-target interactions in vivo by site-specific genome engineering. Nature communications.

[234] Xiong W, Qu Y, Chen H, Qian J (2019). Insight into long noncoding RNA-miRNA-mRNA axes in myocardial ischemia-reperfusion injury: the implications for mechanism and therapy. Epigenomics.

[235] Gu X, Li M, Jin Y, Liu D, Wei F (2017). Identification and integrated analysis of differentially expressed lncRNAs and circRNAs reveal the potential ceRNA networks during PDLSC osteogenic differentiation. BMC genetics.

[236] Mohanapriya R, Akshaya R, Selvamurugan N (2021). A regulatory role of circRNA-miRNA-mRNA network in osteoblast differentiation. Biochimie.

[237] Archer K, Broskova Z, Bayoumi A, Teoh J, Davila A, Tang Y (2015). Long Non-Coding RNAs as Master Regulators in Cardiovascular Diseases. International journal of molecular sciences.

[238] Chen XB, Li W, Chu AX (2019). MicroRNA-133a inhibits gastric cancer cells growth, migration, and epithelial-mesenchymal transition process by targeting presenilin 1. J Cell Biochem.

[239] Wang S, Jun J, Cong L, Du L, Wang C (2021). miR-328-3p, a Predictor of Stroke, Aggravates the Cerebral Ischemia-Reperfusion Injury. Int J Gen Med.

[240] Wang K, Liu CY, Zhang XJ, Feng C, Zhou LY, Zhao Y (2015). miR-361-regulated prohibitin inhibits mitochondrial fission and apoptosis and protects heart from ischemia injury. Cell Death Differ.

[241] Zhou ZW, Ren X, Zheng LJ, Li AP, Zhou WS (2022). LncRNA NEAT1 ameliorate ischemic stroke via promoting Mfn2 expression through binding to Nova and activates Sirt3. Metab Brain Dis.

[242] Ni X, Su Q, Xia W, Zhang Y, Jia K, Su Z (2020). Knockdown lncRNA NEAT1 regulates the activation of microglia and reduces AKT signaling and neuronal apoptosis after cerebral ischemic reperfusion. Sci Rep.

[243] Li F, Zhao H, Li G, Zhang S, Wang R, Tao Z (2020). Intravenous antagomiR-494 lessens brain-infiltrating neutrophils by increasing HDAC2-mediated repression of multiple MMPs in experimental stroke. Faseb j.

[244] Giebel B, Hermann DM (2019). Identification of the right cell sources for the production of therapeutically active extracellular vesicles in ischemic stroke. Ann Transl Med.

[245] Wang C, Borger V, Sardari M, Murke F, Skuljec J, Pul R (2020). Mesenchymal Stromal Cell-Derived Small Extracellular Vesicles Induce Ischemic Neuroprotection by Modulating Leukocytes and Specifically Neutrophils. Stroke.

[246] Gimona M, Brizzi MF, Choo ABH, Dominici M, Davidson SM, Grillari J (2021). Critical considerations for the development of potency tests for therapeutic applications of mesenchymal stromal cell-derived small extracellular vesicles. Cytotherapy.

[247] Trivedi M, Talekar M, Shah P, Ouyang Q, Amiji M (2016). Modification of tumor cell exosome content by transfection with wt-p53 and microRNA-125b expressing plasmid DNA and its effect on macrophage polarization. Oncogenesis.

[248] Que R, Ding G, Chen J, Cao L (2013). Analysis of serum exosomal microRNAs and clinicopathologic features of patients with pancreatic adenocarcinoma. World journal of surgical oncology.

[249] Su J, Li J, Yu Q, Wang J, Li X, Yang J (2020). Exosomal miRNAs as potential biomarkers for acute myocardial infarction. IUBMB life.

[250] Li Z, Yanfang W, Li J, Jiang P, Peng T, Chen K (2018). Tumor-released exosomal circular RNA PDE8A promotes invasive growth via the miR-338/MACC1/MET pathway in pancreatic cancer. Cancer letters.

[251] Huang W, Liu X, Cao J, Meng F, Li M, Chen B (2015). miR-134 regulates ischemia/reperfusion injury-induced neuronal cell death by regulating CREB signaling. J Mol Neurosci.

[252] Zhou J, Chen L, Chen B, Huang S, Zeng C, Wu H (2018). Increased serum exosomal miR-134 expression in the acute ischemic stroke patients. BMC neurology.

[253] Bjørnetrø T, Redalen K, Meltzer S, Thusyanthan N, Samiappan R, Jegerschöld C (2019). An experimental strategy unveiling exosomal microRNAs 486-5p, 181a-5p and 30d-5p from hypoxic tumour cells as circulating indicators of high-risk rectal cancer. Journal of extracellular vesicles.

[254] Zhang R, Xia Y, Wang Z, Zheng J, Chen Y, Li X (2017). Serum long non coding RNA MALAT-1 protected by exosomes is up-regulated and promotes cell proliferation and migration in non-small cell lung cancer. Biochemical and biophysical research communications.

[255] Rong F, Liu L, Zou C, Zeng J, Xu Y (2020). MALAT1 Promotes Cell Tumorigenicity Through Regulating miR-515-5p/EEF2 Axis in Non-Small Cell Lung Cancer. Cancer management and research.

[256] Wang X, Luo G, Zhang K, Cao J, Huang C, Jiang T (2018). Hypoxic Tumor-Derived Exosomal miR-301a Mediates M2 Macrophage Polarization via PTEN/PI3Kγ to Promote Pancreatic Cancer Metastasis. Cancer research.

[257] Zhou D, Gu J, Wang Y, Wu H, Cheng W, Wang Q (2021). Long non-coding RNA NEAT1 transported by extracellular vesicles contributes to breast cancer development by sponging microRNA-141-3p and regulating KLF12. Cell & bioscience.

[258] Zheng M, Liu X, Han R, Yuan W, Sun K, Zhong J (2020). Circulating exosomal long non-coding RNAs in patients with acute myocardial infarction. Journal of cellular and molecular medicine.

[259] Sun L, Zhu W, Zhao P, Wang Q, Fan B, Zhu Y (2020). Long noncoding RNA UCA1 from hypoxia-conditioned hMSC-derived exosomes: a novel molecular target for cardioprotection through miR-873-5p/XIAP axis. Cell death & disease.

[260] Chen Z, Yan Y, Wu J, Qi C, Liu J, Wang J (2020). Expression level and diagnostic value of exosomal NEAT1/miR-204/MMP-9 in acute ST-segment elevation myocardial infarction. IUBMB life.

[261] Ling H, Guo Z, Du S, Liao Y, Li Y, Ding C (2020). Serum exosomal miR-122-5p is a new biomarker for both acute coronary syndrome and underlying coronary artery stenosis. Biomarkers: biochemical indicators of exposure, response, and susceptibility to chemicals.

[262] Ling H, Guo Z, Shi Y, Zhang L, Song C (2020). Serum Exosomal MicroRNA-21, MicroRNA-126, and PTEN Are Novel Biomarkers for Diagnosis of Acute Coronary Syndrome. Frontiers in physiology.

[263] Wang W, Li D, Li R, Zhou X, Yu D, Lan X (2018). Diagnosis of Hyperacute and Acute Ischaemic Stroke: The Potential Utility of Exosomal MicroRNA-21-5p and MicroRNA-30a-5p. Cerebrovascular diseases (Basel, Switzerland).

[264] Chen Y, Song Y, Huang J, Qu M, Zhang Y, Geng J (2017). Increased Circulating Exosomal miRNA-223 Is Associated with Acute Ischemic Stroke. Frontiers in neurology.

[265] Li D, Liu J, Wang W, Li R, Yu D, Lan X (2017). Plasma Exosomal miR-422a and miR-125b-2-3p Serve as Biomarkers for Ischemic Stroke. Current neurovascular research.

[266] Ji Q, Ji Y, Peng J, Zhou X, Chen X, Zhao H (2016). Increased Brain-Specific MiR-9 and MiR-124 in the Serum Exosomes of Acute Ischemic Stroke Patients. PloS one.

[267] Xu H, Chen Y, Dong X, Wang X (2018). Serum Exosomal Long Noncoding RNAs ENSG00000258332.1 and LINC00635 for the Diagnosis and Prognosis of Hepatocellular Carcinoma. Cancer Epidemiol Biomarkers Prev.

[268] Lener T, Gimona M, Aigner L, Borger V, Buzas E, Camussi G (2015). Applying extracellular vesicles based therapeutics in clinical trials - an ISEV position paper. J Extracell Vesicles.

[269] Jia X, Wei L, Zhang Z (2021). NEAT1 Overexpression Indicates a Poor Prognosis and Induces Chemotherapy Resistance via the miR-491-5p/SOX3 Signaling Pathway in Ovarian Cancer. Front Genet.

[270] Lopez MS, Morris-Blanco KC, Ly N, Maves C, Dempsey RJ, Vemuganti R (2021). MicroRNA miR-21 Decreases Post-stroke Brain Damage in Rodents. Transl Stroke Res.

[271] Liu W, Chen X, Zhang Y (2016). Effects of microRNA-21 and microRNA-24 inhibitors on neuronal apoptosis in ischemic stroke. Am J Transl Res.

[272] Feng H, Xie B, Zhang Z, Yan J, Cheng M, Zhou Y (2021). MiR-135a Protects against Myocardial Injury by Targeting TLR4. Chem Pharm Bull (Tokyo).

